# Immunopeptidomics-Guided Warehouse Design for Peptide-Based Immunotherapy in Chronic Lymphocytic Leukemia

**DOI:** 10.3389/fimmu.2021.705974

**Published:** 2021-07-08

**Authors:** Annika Nelde, Yacine Maringer, Tatjana Bilich, Helmut R. Salih, Malte Roerden, Jonas S. Heitmann, Ana Marcu, Jens Bauer, Marian C. Neidert, Claudio Denzlinger, Gerald Illerhaus, Walter Erich Aulitzky, Hans-Georg Rammensee, Juliane S. Walz

**Affiliations:** ^1^ Clinical Collaboration Unit Translational Immunology, German Cancer Consortium (DKTK), Department of Internal Medicine, University Hospital Tübingen, Tübingen, Germany; ^2^ Department of Immunology, Institute for Cell Biology, University of Tübingen, Tübingen, Germany; ^3^ Cluster of Excellence iFIT (EXC2180) “Image-Guided and Functionally Instructed Tumor Therapies”, University of Tübingen, Tübingen, Germany; ^4^ Department of Hematology, Oncology, Clinical Immunology and Rheumatology, University Hospital Tübingen, Tübingen, Germany; ^5^ Department of Neurosurgery, Cantonal Hospital St. Gallen, St. Gallen, Switzerland; ^6^ Marienhospital, Stuttgart, Germany; ^7^ Clinic for Hematology and Oncology, Klinikum Stuttgart, Stuttgart, Germany; ^8^ Department of Hematology, Oncology and Palliative Medicine, Robert-Bosch-Krankenhaus Stuttgart, Stuttgart, Germany; ^9^ German Cancer Consortium (DKTK) and German Cancer Research Center (DKFZ), partner site Tübingen, Tübingen, Germany; ^10^ Dr. Margarete Fischer-Bosch Institute of Clinical Pharmacology and Robert Bosch Center for Tumor Diseases (RBCT), Stuttgart, Germany

**Keywords:** peptide vaccines, HLA peptides, chronic lymphocytic leukemia, mass spectrometry, peptide warehouse, immunopeptidomics, immunotherapy

## Abstract

Antigen-specific immunotherapies, in particular peptide vaccines, depend on the recognition of naturally presented antigens derived from mutated and unmutated gene products on human leukocyte antigens, and represent a promising low-side-effect concept for cancer treatment. So far, the broad application of peptide vaccines in cancer patients is hampered by challenges of time- and cost-intensive personalized vaccine design, and the lack of neoepitopes from tumor-specific mutations, especially in low-mutational burden malignancies. In this study, we developed an immunopeptidome-guided workflow for the design of tumor-associated off-the-shelf peptide warehouses for broadly applicable personalized therapeutics. Comparative mass spectrometry-based immunopeptidome analyses of primary chronic lymphocytic leukemia (CLL) samples, as representative example of low-mutational burden tumor entities, and a dataset of benign tissue samples enabled the identification of high-frequent non-mutated CLL-associated antigens. These antigens were further shown to be recognized by pre-existing and *de novo* induced T cells in CLL patients and healthy volunteers, and were evaluated as pre-manufactured warehouse for the construction of personalized multi-peptide vaccines in a first clinical trial for CLL (NCT04688385). This workflow for the design of peptide warehouses is easily transferable to other tumor entities and can provide the foundation for the development of broad personalized T cell-based immunotherapy approaches.

## Introduction

The breakthrough clinical success of T cell-based immunotherapy approaches, including immune checkpoint inhibitors, CAR T cells, bispecific antibodies, and adoptive T cell transfer, has recently revolutionized the treatment of solid tumors and hematological malignancies. However, some patients do not respond to available therapies at all, others only temporarily calling for further efficacy improvement of T cell-centered immunotherapies. Peptide-based approaches, which rely on the specific immune recognition of tumor-associated human leukocyte antigen (HLA)-presented peptides, represent promising alternatives with low side effects. Targeting mutation-derived neoepitopes by vaccination in melanoma – a high-mutational burden tumor entity – has demonstrated immunogenicity and first clinical efficacy (1). However, only a minority of mutations at the DNA level is translated and naturally processed to HLA-presented neoepitopes targetable for T cells (2, 3). Non-mutated tumor-associated antigens, arising through differential gene expression or protein processing in tumor cells, might supplement or even substitute neoepitope targeting in low-mutational burden malignancies. Whereas a huge number of clinical studies have shown immune responses, including strong CD8^+^ T cell responses, to vaccines targeting non-mutated tumor antigens, so far, all performed trials failed to show meaningful clinical results (4–10). Nonetheless, several studies showed a pathophysiological relevance of these tumor antigens, correlating spontaneous, pre-existing as well as immune checkpoint inhibitor-induced T cell responses, targeting non-mutated tumor antigens with improved clinical outcome of malignancies (11–14). Therefore, the usage of novel technologies and methods, to unravel and resolve the underlying issues and limitations of non-mutated antigen-based vaccines, might enable that T cell responses, induced or boosted by these vaccines, may result in clinical effectiveness in the future (15).

One issue of former peptide-vaccine trials was the selection of non-mutated tumor antigens that were not proven to be naturally presented on the tumor cell surface of the individual patients. For multiple of the applied “classical” tumor antigens novel analyses showed a distorted correlation of tumor-associated presentation on mRNA level, and limited or even lacking presentation on cell surface HLA (16–22).

In recent years, characterization of naturally presented mutated and non-mutated tumor antigens was refined using direct, mass spectrometry-based analyses of the entirety of HLA ligands termed the immunopeptidome (13, 20–25). While mass spectrometric identification of neoepitopes remains rare with inter-individual heterogeneity and requires a time- and cost-intensive fully personalized vaccine design (1), distinct panels of high-frequent non-mutated tumor-associated antigens have been previously identified (13, 20–24). Such panels could enable the construction of off-the-shelf pre-manufactured peptide warehouses for the design of broadly applicable personalized therapeutics, such as multi-peptide vaccines or products for adoptive T cell transfer, that could be assembled based on patient-individual characteristics. To evaluate this concept in chronic lymphocytic leukemia (CLL) as representative example of an immunogenic, low-mutational burden malignancy (26, 27), we here developed a widely applicable workflow for the immunopeptidomics-guided design of such a warehouse, that provides the basis for the selection of personalized multi-peptide vaccine cocktails for a first-in-man clinical trial (NCT04688385).

## Materials and Methods

### Patients and Blood Samples

Peripheral blood mononuclear cells (PBMCs) from CLL patients as well as PBMCs from healthy volunteers (HVs) were isolated by density gradient centrifugation and stored at -80°C until further use for subsequent HLA immunoprecipitation or T cell-based assays. For immunopeptidome analysis, PBMCs from CLL patients with white blood cell counts ≥ 20 000 per µl were used. Informed consent was obtained in accordance with the Declaration of Helsinki protocol. The study was performed according to the guidelines of the local ethics committees (373/2011B02, 454/2016B02, 406/2019BO2). HLA typing of CLL patient samples was carried out by the Department of Hematology and Oncology, Tübingen, Germany. Patient characteristics of the immunopeptidome cohort (n = 61) are provided in **Supplementary Table 1**. Sample characteristics of the immunogenicity cohort (n = 51) are provided in **Supplementary Table 2**.

### Isolation of HLA Ligands

HLA class I and HLA class II molecules were isolated by standard immunoaffinity purification (28) using the pan-HLA class I-specific monoclonal antibody W6/32, the pan-HLA class II-specific monoclonal antibody Tü-39, and the HLA-DR-specific monoclonal antibody L243 (all produced in-house) to extract HLA ligands.

### Analysis of HLA Ligands by Liquid Chromatography-Coupled Tandem Mass Spectrometry (LC-MS/MS)

Isolated peptide samples were analyzed as described previously (13, 29). Peptides were separated by nanoflow high-performance liquid chromatography. Eluted peptides were analyzed in an online-coupled LTQ Orbitrap XL or Orbitrap Fusion Lumos mass spectrometer (both Thermo Fisher, Waltham, Massachusetts, USA).

### Data Processing

Data processing was performed as described previously (13, 29). The Proteome Discoverer (v1.3, Thermo Fisher) was used to integrate the search results of the SequestHT search engine (University of Washington) (30) against the human proteome (Swiss-Prot database, 20 279 reviewed protein sequences, September 27th 2013) accompanied with recurrent somatic CLL-associated mutations in 61 different proteins (**Supplementary Table 3**) as described in the literature (31, 32) and assigned in the COSMIC database (http://cancer.sanger.ac.uk) (33) without enzymatic restriction. Precursor mass tolerance was set to 5 ppm. Fragment mass tolerance was set to 0.5 Da for ion trap spectra (LTQ Orbitrap XL) and 0.02 Da for orbitrap spectra (Orbitrap Fusion Lumos). Oxidized methionine was allowed as dynamic modification. The false discovery rate (FDR) estimated by the Percolator algorithm 2.04 (34) was limited to 5% for HLA class I- and 1% for HLA class II-presented peptides. HLA class I annotation was performed using SYFPEITHI 1.0 (35) and NetMHCpan 4.0 (36, 37).

### Peptide Synthesis

Peptides were produced by the peptide synthesizer Liberty Blue (CEM, Kamp-Lintfort, Germany) using the 9-fluorenylmethyl-oxycarbonyl/tert-butyl strategy (38). Peptides for the peptide warehouse were selected with regard to a subsequent good manufacturing practice (GMP) and manufacturing license conform production process, including peptide length restriction of 20 amino acids, avoiding cysteine-containing peptides as well as peptides with histidine or proline at the C-terminus.

### Spectrum Validation

Spectrum validation of the experimentally eluted peptides was performed by computing the similarity of the spectra with corresponding synthetic peptides measured in a complex matrix. The spectral correlation was calculated between the MS/MS spectra of the eluted and the synthetic peptide (39).

### Amplification of Peptide-Specific T Cells and Interferon-γ (IFN-γ) Enzyme-Linked Immunospot (ELISpot) Assay

PBMCs from CLL patients were treated with 1 µg/ml anti-human PD-1 monoclonal antibody (CD279, #14-2799-80, Invitrogen, Carlsbad, CA, USA) and 1 µg/ml anti-human CTLA-4 monoclonal antibody (CD152, #16-1529-82, Invitrogen) for one hour before pulsing with 1 µg/ml or 5 µg/ml per HLA class I or HLA class II peptide, respectively. Negative control peptides with the respective HLA restrictions were also used for stimulation: YLLPAIVHI for HLA-A*02 (source protein: DDX5_HUMAN), KYPENFFLL for HLA-A*24 (source protein: PP1G_HUMAN), TPGPGVRYPL for HLA-B*07 (source protein: NEF_HV1BR) and ETVITVDTKAAGKGK for HLA class II (source protein: FLNA_HUMAN). Cells were cultured for 12 days adding 20 U/ml IL-2 (Novartis, Basel, Switzerland) on days 2, 5, and 7 (13, 22). Peptide-stimulated PBMCs were analyzed by IFN-γ ELISpot assay on day 12 (23, 40). Spots were counted using an ImmunoSpot S6 analyzer (CTL, Cleveland, OH, USA) and T cell responses were considered positive when > 10 spots/500 000 cells were counted, and the mean spot count was at least three-fold higher than the mean spot count of the negative control.

### Refolding

Biotinylated HLA:peptide complexes were manufactured as described previously (41) and tetramerized using PE-conjugated streptavidin (Invitrogen) at a 4:1 molar ratio.

### Induction of Peptide-Specific CD8^+^ T Cells With Artificial Antigen-Presenting Cells (aAPCs)

Priming of peptide-specific cytotoxic T lymphocytes was conducted using aAPCs as described previously  (42). In detail, 800 000 streptavidin-coated microspheres (Bangs Laboratories, Fishers, Indiana, USA) were loaded with 200 ng biotinylated HLA:peptide monomer and 600 ng biotinylated anti-human CD28 monoclonal antibody (clone 9.3, in-house production). CD8^+^ T cells were cultured with 4.8 U/µl IL-2 (R&D Systems, Minneapolis, MN, USA) and 1.25 ng/ml IL-7 (PromoKine, Heidelberg, Germany). Weekly stimulation with aAPCs (200 000 aAPCs per 1 × 10^6^ CD8^+^ T cells) and 5 ng/ml IL-12 (PromoKine) was performed four times.

### Cytokine and Tetramer Staining

The functionality of peptide-specific CD8^+^ T cells was analyzed by intracellular cytokine staining (ICS) as described previously (40, 43). Cells were pulsed with 10 µg/ml of individual peptide and incubated with FITC anti-human CD107a (BioLegend, San Diego, CA, USA) for one hour before incubating with 10 μg/ml Brefeldin A (Sigma-Aldrich, Saint Louis, MO, USA) and 10 μg/ml GolgiStop (BD, Franklin Lakes, NJ, USA) for 12-16 h. Staining was performed using aqua fluorescent reactive dye (Invitrogen), Cytofix/Cytoperm (BD), PE-Cy7 anti-human CD8 (Beckman Coulter, Brea, CA, USA), Pacific Blue anti-human tumor necrosis factor (TNF), and PE anti-human IFN-γ (Biolegend) monoclonal antibodies. PMA and ionomycin (Sigma-Aldrich) served as positive control. Negative control peptides were used as described for the ELISpot assays. The frequency of peptide-specific CD8^+^ T cells after aAPC-based priming was determined by PE-Cy7 anti-human CD8 monoclonal antibody and HLA:peptide tetramer-PE staining. Cells of the same donor primed with an irrelevant control peptide and stained with the tetramer containing the test peptide were used as negative control. The priming was considered successful if the frequency of peptide-specific CD8^+^ T cells was ≥ 0.1% of CD8^+^ T cells within the viable single cell population and at least three-fold higher than the frequency of peptide-specific CD8^+^ T cells in the negative control. The same evaluation criteria were applied for ICS results. Samples were analyzed on a FACS Canto II cytometer (BD).

### Software and Statistical Analysis

An in-house Python script was used for the calculation of FDRs of CLL-associated peptides at different presentation frequencies (13). Overlap analysis was performed using BioVenn (44). The population coverage of HLA allotypes was calculated by the IEDB population coverage tool (www.iedb.org) (45, 46). Flow cytometric data was analyzed using FlowJo 10.0.8 (Treestar, Ashland, Oregon, USA). All figures and statistical analyses were generated using GraphPad Prism 9.0.2 (GraphPad Software, San Diego, CA, USA).

## Results

### Mass Spectrometry-Based Identification of Naturally Presented CLL-Associated Antigens

**Figure 1:**
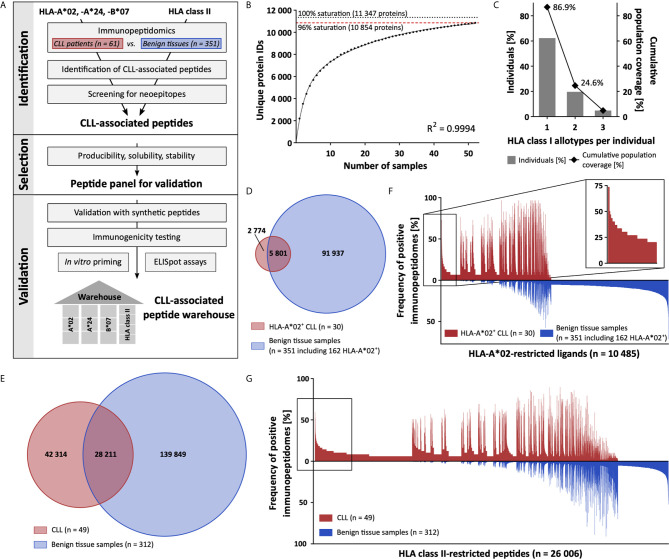
Comparative immunopeptidome profiling identifies CLL-associated antigens. **(A)** Mass spectrometry-based workflow for the design of a CLL-associated immunopeptidome-derived peptide warehouse. **(B)** Saturation analysis of source proteins of HLA class I-presented peptides. Number of unique source protein identifications shown as a function of cumulative immunopeptidome analysis of CLL samples (n = 52). Exponential regression allowed for the robust calculation of the maximum attainable number of different source protein identifications (dotted line). The dashed red line depicts the source proteome coverage achieved in the CLL cohort. **(C)** HLA-A*02, -A*24, and -B*07 allotype coverage within the CLL cohort (n = 61). The frequencies of individuals within the CLL cohort carrying up to three HLA allotypes (x-axis) are indicated as gray bars on the left y-axis. The cumulative percentage of population coverage is depicted as black dots on the right y-axis. **(D, E)** Overlap analysis of **(D)** HLA-A*02- and **(E)** HLA class II-restricted peptide identifications of primary CLL samples (D, n = 30; E, n = 49) and benign tissue samples (D, n = 351 including 162 HLA-A*02^+^; E, n = 312). **(F, G)** Comparative immunopeptidome profiling of **(F)** HLA-A*02- and **(G)** HLA class II-presented peptides based on the frequency of HLA-restricted presentation in immunopeptidomes of CLL and benign tissue samples. Frequencies of positive immunopeptidomes for the respective HLA-presented peptides (x-axis) are indicated on the y-axis. To allow for better readability, HLA-presented peptides identified in < 5% of the samples within the respective cohort were not depicted. The box on the left highlights the subset of CLL-associated antigens that show CLL-exclusive high-frequent presentation. IDs, identifications.

In the first step of our workflow for the definition of an off-the-shelf peptide warehouse (**Figure 1A**), we comprehensively mapped the immunopeptidome of 61 primary CLL samples (n = 52 for HLA class I, n = 49 for HLA class II, **Supplementary Table 1**). We identified a total of 58 554 unique HLA class I ligands representing 10 854 source proteins, obtaining 96% of the estimated maximum attainable source protein coverage (**Figure 1B**). The number of identified unique peptides per patient ranged from 527 to 9 530 (mean 3 035, **Supplementary Table 4**). For HLA class II, we identified 70 525 different peptides (range 575-10 392, mean 3 842 per sample, **Supplementary Table 4**) derived from 6 074 source proteins, achieving 85% of maximum attainable coverage (**Supplementary Figure 1A**).

For the identification of broadly applicable non-mutated CLL-associated antigens, we focused on antigens presented by the common allotypes HLA-A*02, -A*24, and -B*07. In total, 87% (53/61, 61% A*02^+^, 28% A*24^+^, 28% B*07^+^) of CLL patients in our cohort carry at least one of the selected HLA class I allotypes (**Figure 1C**). In comparison, 76% of patients included in a previous CLL peptide vaccination trial (NCT02802943), 68% of the European population, and 61% of the world population carry one or more of these HLA allotypes (**Supplementary Figures 1B–D**). Allotype-specific immunopeptidome analysis revealed 8 575 unique HLA-A*02- (range 82-3 723, mean 1 121 per sample), 5 280 unique HLA-A*24- (range 134-1 926, mean 1 042 per sample), and 5 780 unique HLA-B*07-restricted (range 223-2 933, mean 1 140 per sample) peptides derived from 5 020, 3 813, and 3 886 source proteins, achieving 89%, 85%, and 86% of the estimated maximum attainable protein coverage, respectively (**Supplementary Figures 1E–G, Supplementary Table 4**). For comparative immunopeptidome profiling we utilized a dataset of benign hematological and non-hematological (www.hla-ligand-atlas.org) tissue samples (n = 351 for HLA class I, n = 312 for HLA class II) including 162 HLA-A*02^+^, 39 HLA-A*24^+^, and 63 HLA-B*07^+^ samples, comprising 97 738 unique HLA class I- and 168 060 HLA class II-presented peptides. Overlap analysis of the total HLA class I immunopeptidome of the CLL cohort with the benign tissue cohort revealed 23 676 peptides to be exclusively presented on CLL samples and never on benign tissue samples (**Supplementary Figure 1H**). Allotype-specific overlap analysis with the entirety of benign tissue-derived immunopeptidomes revealed 2 774 HLA-A*02-, 1 440 HLA-A*24-, and 1 450 HLA-B*07-presented peptides detected exclusively on CLL samples (**Figure 1D** and **Supplementary Figures 1I, J**). For HLA class II, overlap analysis identified 42 314 peptides to be CLL-exclusive (**Figure 1E**). At a target-definition FDR of < 1%, a total of 393 HLA-A*02-, 168 HLA-A*24-, and 127 HLA-B*07-restricted ligands with allotype-specific representation frequencies up to 73%, 81%, and 60%, respectively, and 3 970 HLA class II-restricted peptides with frequencies up to 59% were identified (**Figures 1F, G** and **Supplementary Figures 1K, L, 2**).

### The Role of Neoantigens in the Immunopeptidome of CLL

**Figure 2:**
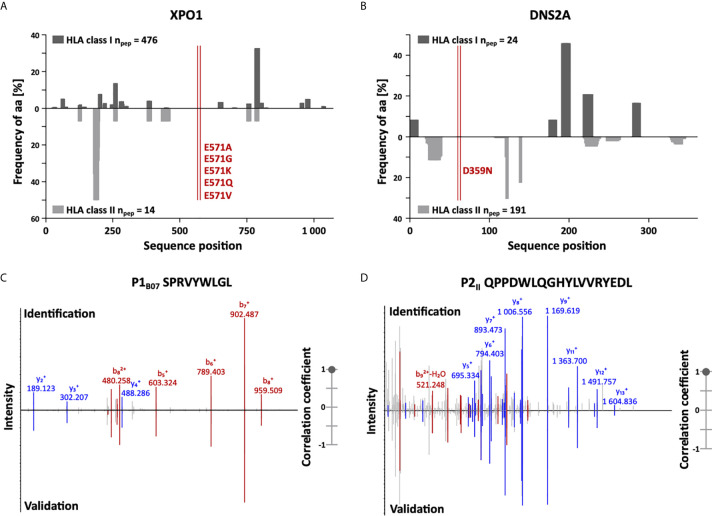
Immunopeptidome coverage of common CLL mutation sites and spectral validation of CLL-associated peptides. **(A, B)** Hotspot and dark spot analysis by HLA class I (above x-axis) and HLA class II (below x-axis) peptide clustering. All identified HLA class I- and HLA class II-presented peptides of the CLL and benign tissue immunopeptidomes were mapped to their amino acid positions within the respective source protein. Representative examples are shown for **(A)** XPO1 and **(B)** DNS2A. Representation frequencies of amino acid counts for the respective amino acid position (x-axis) were calculated and are indicated on the y-axis. The red lines highlight the analyzed mutation sites of recurrent CLL-associated mutations. **(C, D)** Representative examples of the validation of the experimentally eluted **(C)** HLA class I-restricted peptide P1_B07_ and **(D)** the HLA class II-restricted peptide P2_II_ using synthetic isotope-labeled peptides. Comparison of fragment spectra (m/z on the x-axis) of peptides eluted from primary CLL patient samples (identification) to their corresponding synthetic peptides (validation). The spectra of the synthetic peptides are mirrored on the x-axis. Identified b- and y-ions are marked in red and blue, respectively. The calculated spectral correlation coefficients are depicted on the right graph, respectively. aa, amino acid; n_pep_, number of peptides.

In addition to the identification of high-frequent non-mutated tumor-associated peptides, we screened our CLL cohort for naturally presented neoepitopes derived from common CLL-associated point and frameshift mutations (95 point and 3 frameshift mutations within 61 different genes representing 85 different mutation sites, **Supplementary Table 3**). Even though these mutations potentially provide HLA binding motifs for several HLA allotypes, no naturally HLA-presented neoepitopes were identified in our immunopeptidome analyses. Of note, we were able to identify wild-type peptides derived from 57/61 (93%) mutation-bearing proteins in benign and/or CLL immunopeptidomes with an average of 15 and 11 HLA class I- and HLA class II-presented peptides per protein, respectively. However, only 17/85 (20%) mutation sites are covered directly by wild-type peptides as most of the recurrent CLL-associated mutations are located in “dark spots” of the immunopeptidome, defined as protein regions without any detectable HLA-presented peptides (**Figures 2A, B**).

### Definition of Peptide Warehouse

For the setup of a broadly applicable peptide warehouse, we focused on the 532 non-mutated peptides presented by ≥ 20% of (HLA-matched) CLL samples comprising 82 HLA-A*02-, 105 HLA-A*24-, 127 HLA-B*07-, and 218 HLA class II-restricted peptides (**Supplementary Tables 5–8**). For HLA class II, 184/218 (84%) peptides showed length variants (> 50% overlap) that were presented on benign samples and were therefore omitted.

To enable the peptide warehouse production for clinical application, further selection steps, including approval of producibility, solubility, and stability of target antigens under GMP conditions, delineated a 14-peptide panel (**Table 1**) comprising 9 HLA class I- (3 for each allotype) and 5 HLA class II-restricted CLL-exclusive high-frequent target antigens. Experimentally acquired spectra of the selected peptide identifications were validated by comparison of mass spectrometric fragment spectra using isotope-labeled synthetic peptides (**Figures 2C, D** and **Supplementary Figure 3**).

**Table 1:** Immunogenicity of CLL-associated warehouse peptides.

Peptide ID	Sequence	Source protein	HLA restriction	Peptide length	Allotype-specific frequency	T cell response in CLL	*In vitro* CD8^+^ T cell priming in HVs	Functionality of peptide-specific T cells after *in vitro* priming
**P1_A02_**	VIAELPPKV	IGHM	A*02	9	47%	0/19 (0%)	3/3	TNF^+^
**P2_A02_**	ALHRPDVYL	IGHM	A*02	9	40%	0/19 (0%)	3/3	TNF^+^
**P3_A02_**	TLDTSKLYV	RGRF1	A*02	9	37%	0/19 (0%)	3/3	IFN-γ^+^ TNF^+^
**P1_A24_**	GYMPYLNRF	SWP70	A*24	9	56%	0/14 (0%)	3/3	IFN-γ^+^ TNF^+^
**P2_A24_**	KYSKALIDYF	AFF3	A*24	10	50%	0/14 (0%)	2/3	IFN-γ^+^ TNF^+^
**P3_A24_**	RHTGALPLF	SI1L3	A*24	9	50%	0/14 (0%)	3/3	IFN-γ^+^ TNF^+^ CD107a^+^
**P1_B07_**	SPRVYWLGL	CL17A	B*07	9	53%	4/14 (29%)	3/3	IFN-γ^+^ TNF^+^ CD107a^+^
**P2_B07_**	RPSNKAPLL	EHMT1	B*07	9	47%	0/14 (0%)	3/3	IFN-γ^+^ TNF^+^ CD107a^+^
**P3_B07_**	LPRLEALDL	TLR9	B*07	9	40%	4/14 (29%)	3/3	IFN-γ^+^ TNF^+^ CD107a^+^
**P1_ll_**	GSSFFGELFNQNPE	CHST2	class II	14	59%	4/10 (40%)	–	–
**P2_ll_**	QPPDWLQGHYLVVRYEDL	CHST2	class II	18	29%	5/10 (50%)	–	–
**P3_ll_**	YPDRPGWLRYIQRTPYSDG	SGCE	class II	19	27%	2/10 (20%)	–	–
**P4_ll_**	DHAQLVAIKTLKDYNNPQ	ROR1	class II	18	24%	0/10 (0%)	–	–
**P5_ll_**	LLLILRDPSERVLSDY	HS3S1	class II	16	24%	3/10 (30%)	–	–

ID, identification; HVs, healthy volunteers.

### Warehouse Peptides Show Pre-Existing and *De Novo* Inducible Immune Responses in CLL Patients and HVs

**Figure 3:**
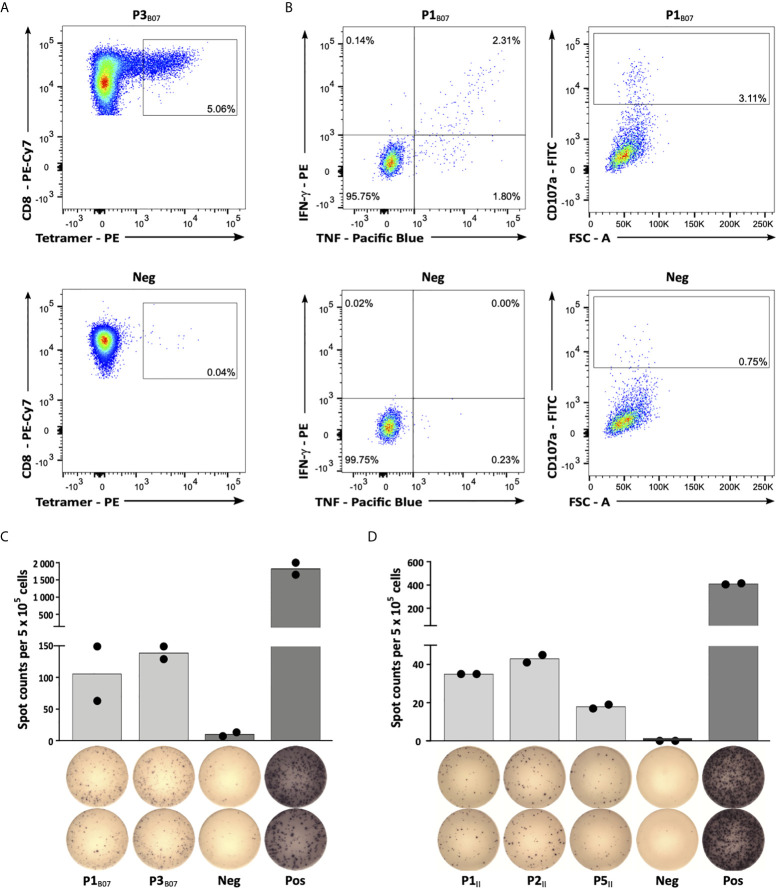
Immunogenicity analyses of CLL-associated peptides. **(A)** Representative example of P3_B07_-specific tetramer staining of CD8^+^ T cells after 4 cycles of aAPC-based in *vitro* priming. Graphs show single, viable cells stained for CD8 and PE-conjugated multimers of indicated specificity. The upper panel displays P3_B07_-tetramer staining of T cells primed with P3_B07_. The lower panel (negative control) depicts P3_B07_-tetramer staining of T cells from the same donor primed with an HLA-matched irrelevant control peptide. **(B)** Functional characterization of induced P1_B07_-specific CD8^+^ T cells after in *vitro* aAPC-based priming by intracellular cytokine (IFN-γ, TNF) and degranulation marker (CD107a) staining. Representative example of IFN-γ and TNF production as well as CD107a expression after stimulation with the peptide P1_B07_ compared to an HLA-matched negative control peptide. **(C, D)** Representative examples of preexisting T cell responses to **(C)** HLA-B*07- and **(D)** HLA class II-restricted peptides as evaluated by IFN-γ ELISpot assays after 12-day in *vitro* expansion using PBMC samples of CLL patients (C, UPN064; D, UPN066). Data are presented as scatter dot plot with mean. FSC, forward scatter; Neg, negative control; Pos, positive control.

In the final step, selected peptide targets were analyzed for their immunogenicity, *i.e.*, their potential to induce antigen-specific T cell responses. Using aAPC-based *in vitro* priming of naive CD8^+^ T cells of HLA-matched HVs, we confirmed induction of peptide-specific CD8^+^ T cells for all 9 HLA class I peptides in at least 2/3 HVs (**Figure 3A**, **Supplementary Figures 4A, B** and **Table 1**). Intracellular cytokine and degranulation marker staining revealed induction of multifunctional peptide-specific T cells for 7/9 (78%) peptides (**Figure 3B**, **Supplementary Figures 4C, D** and **Table 1**). Moreover, IFN-γ ELISpot assays, using PBMCs from HLA-matched CLL patients, revealed preexisting peptide-specific memory T cells targeting 2/9 (22%) and 4/5 (80%) HLA class I- and HLA class II-restricted warehouse peptides in up to 29% and 50% of CLL patient samples, respectively (**Figures 3C, D**, **Supplementary Figure 5** and **Table 1**). In total, we validated 13/14 (93%) of the preselected naturally presented CLL-associated HLA class I- and HLA class II-restricted peptides as immunogenic, with either pre-existing or *de novo* inducible immune responses, unveiling these as ideal targets for peptide-based immunotherapy approaches.

## Discussion

We present a novel workflow for the immunopeptidomics-guided design of off-the-shelf peptide warehouses, here applied to CLL as a representative of low-mutational burden tumor entities. The mass spectrometry-based characterization of high-frequent, non-mutated, tumor-associated, and immunogenic antigens naturally presented on primary CLL samples enables the development of a personalized peptide-based immunotherapy in a time- and cost-effective manner, and can be easily transferred to other tumor entities.

The treatment landscape for CLL has faced profound changes with the development and clinical success of targeted therapies in the past years (47, 48), including the Bruton’s tyrosine kinase (BTK) inhibitor ibrutinib (49). However, beside the combination of anti-CD20 antibodies and venetoclax, all novel substances require continuous treatment bearing the risk of therapy resistance and accumulation of side effects. Current efforts are now focusing on the further and earlier elimination of minimal residual disease (MRD) to allow for reduced treatment duration and the therewith associated side effects, as well as the achievement of long-lasting remission and potential cure in the future. The favorable immune effector-to-target cell ratios in the MRD setting and the immunogenicity of CLL (26, 27) suggest that this malignancy might be effectively targeted by peptide-based immunotherapy.

For the clinical application of such approaches three different strategies have been proposed. (i) Stratification, applying an invariant drug product to every patient, seems unsuitable due to the patient-individual HLA allotypes. Therefore, such an approach must focus on very common HLA allotypes thereby excluding a substantial proportion of patients. In addition, as shown by us and others, even in allotype-matched patients of the same entity, presentation of tumor-associated peptides is not given in 100% of tumors, calling for more personalized approaches of target selection (21, 50). However, the broad applicability of (ii) completely individualized peptide vaccine concepts is hampered, since these are based on time- and cost-intensive patient-specific identification and selection processes and on-demand *de novo* drug production (1, 5, 51). The approach of (iii) peptide warehouse design, comprising a collection of pre-defined and pre-manufactured high-frequent tumor-associated peptides, enables the subsequent composition of patient-specific vaccine cocktails based on individual characteristics (5, 10, 52).

In recent years, multiple peptide vaccination approaches were focused on the targeting of neoepitopes from tumor mutations as prime tumor-specific targets. For tumor entities with high mutational burden, such as melanoma, high immunogenicity and first signs of clinical efficacy have been demonstrated (1, 53). However, only a minority of mutations at the DNA sequence level is translated and processed to naturally presented HLA-restricted neoepitopes that can be targeted by T cells (2, 16, 54, 55). This is in line with our data demonstrating the lack of naturally presented neoantigens in the immunopeptidome of CLL. Together with recent immunopeptidomic studies (16, 21, 56) we showed that HLA-presented peptides are not randomly distributed across protein sequences but rather show “hotspot” locations of presentation. Most of the recurrent CLL-associated mutations are located in “dark spots” of the immunopeptidome, defined as protein regions without any detectable HLA-presented peptides, explaining the rare detection of neoepitopes especially in low-mutational burden entities. The underlying reasons for the occurrence of such hotspots and corresponding dark spots still remain ambiguous but might include differential proteasomal cleavage, peptide processing, and HLA-binding (16, 56, 57). Thus, the role of neoantigen-based T cell responses in tumor entities with low-mutational burden remains obscure, calling for the application of alternative targets in peptide-based immunotherapy approaches (1–3). Non-mutated tumor peptides, arising through altered gene expression or protein processing in the tumor cells have been suggested as vaccine targets for many years (4–10, 13, 58, 59). However, although numerous clinical trials reported peptide-specific T cell induction upon vaccination with non-mutated tumor antigens, no correlation with clinical activity was shown and no meaningful clinical results were achieved (4–10). Nevertheless, there are two main points that prompt us and others, despite former disappointing clinical data, to use novel technologies and methods to unravel and resolve the underlying issues and limitations of non-mutated antigen-based vaccines: (i) Several studies report on the existence of spontaneous preexisting T cell responses targeting non-mutated tumor antigens and their correlation with beneficial clinical outcome, suggesting a pathophysiological relevance of these immune responses *in vivo* (11–14) and (ii) recent data show that immune checkpoint inhibitor-mediated T cell responses are not only targeting neoepitopes but also non-mutated tumor antigens (60–62).

Several unmet issues that hamper the development of effective peptide vaccines have been identified in recent years and need to be considered and addressed during future peptide vaccine design. These include target antigen selection, time points of application, and selection of combinatorial drugs (15, 63). Within this study we aimed to address one obvious and often discussed issue of former vaccination trials, where peptide selection included non-mutated tumor antigens that were never proven to be naturally presented on the tumor cell surface of the individual patients. For multiple of the applied “classical” tumor antigens novel analyses showed a distorted correlation of tumor-associated presentation on mRNA level, and limited or even lacking presentation on the immunopeptidome level (16–22), highlighting that the immunopeptidome is an independent complex layer formed by the antigen presentation machinery, and does not necessarily mirror the transcriptome or proteome. Therefore, it is essential to use direct methods of peptide target identification (64). This can be realized by mass spectrometry-based analysis of the entirety of naturally presented HLA ligands, termed the HLA ligandome or immunopeptidome of cancer cells (65). In recent years, we and others worked intensively on the characterization of such naturally presented tumor-associated peptides based on the direct isolation of HLA class I- and class II-presented ligands from tumor cells and the subsequent identification by mass spectrometry (21–24, 66, 67). Our approach allowed the identification of distinct panels of high-frequent non-mutated tumor peptides across multiple donors.

In addition to cytotoxic CD8^+^ T cells, CD4^+^ T cells play important direct and indirect roles in anti-cancer immunity (68) and therefore are indispensable for vaccination approaches. Consequently, the here described workflow comprises target selection of multiple HLA class I- and HLA class II-presented peptides to prevent antigen loss and reduce the risk of immune escape, which often occurs under therapeutic pressure (69, 70). Furthermore, the promiscuous binding motifs of HLA class II molecules enable a broad allotype-independent application of these peptides.

Moreover, the detection of preexisting memory T cell responses targeting our warehouse peptides underscores the pathophysiological relevance of our selected antigen targets for immune surveillance in CLL.

Further limitations of peptide vaccines in general, and in particular of non-mutated antigen vaccine peptides, comprise tumor evasion, immune-editing and immune cell exhaustion (71). Increased numbers of regulatory T cells (T_reg_) have been associated with a worse outcome of vaccination, both in mice and patients (72, 73). In addition, peripheral tolerance limits the available T cell repertoire capable of recognizing cancer cells with high affinity. T cell recruitment is often impaired by an immunosuppressive tumor environment (74) and the aberrant tumor vasculature actively suppresses the access of cancer-specific T cells (75), which limits therapeutic vaccine efficacy. Therefore, combinatorial approaches to further improve vaccine-induced effects are currently being investigated. These include strategies to deplete or modulate T_regs_ (7, 76–80) as well as the development of modified, so-called heteroclitic, vaccine peptides to enhance low-affinity T cells (81, 82). Furthermore, to overcome limited T cell function and recruitment, combinatorial approaches with immune checkpoint inhibitors (83, 84) as well as with direct or indirect microenvironment modifiers, such as MEK or PARP inhibitors as well as VEGF-targeting antibody- or inhibitor-based therapies, are already evaluated (85). The importance of a rational selection of combinatorial drugs was recently demonstrated in a phase III peptide vaccination study (86), which failed to confirm the vaccine-induced immune responses and clinical outcome reported in the preceding phase II trial (7). Here, the combination of the peptide vaccine with the tyrosine kinase inhibitor sunitinib, for which a negative impact on T cell responses was described (87), was suggested as an underlying reason. In contrast, a supporting and positive effect on T cell functionality was proven for other tyrosine kinase inhibitors, such as ibrutinib (88–90), suggesting this BTK inhibitor as a potential combination drug for peptide vaccines in CLL patients.

Together, this study presents a mass spectrometry-based workflow for the design of an immunopeptidome-derived off-the-shelf CLL-associated peptide warehouse, which is currently being evaluated within a first personalized multi-peptide vaccine trial in combination with the novel adjuvant XS15 (91) in CLL patients under ibrutinib treatment (iVAC-XS15-CLL01, NCT04688385). Integrating next generation developments and insights, in terms of antigen selection, interaction of tumor cells with the immune system and rational selection of combination therapies, we aim to contribute a further step on the way to clinically effective peptide vaccinations with this study. Our warehouse design concept is further easily transferable to other tumor entities, enabling the construction of broadly applicable peptide warehouses, which provide the foundation for the development of time- and cost-effective personalized T cell-based immunotherapy approaches.

## Data Availability Statement

The mass spectrometry data have been deposited to the ProteomeXchange Consortium (http://proteomecentral.proteomexchange.org) via the PRIDE (92) partner repository with the dataset identifier PXD024871.

## Ethics Statement

The studies involving human participants were reviewed and approved by Ethics committee of the University Hospital Tübingen. The patients/participants provided their written informed consent to participate in this study.

## Author Contributions

AN, YM, H-GR, and JW designed the study. AN, AM, and JB performed immunopeptidome experiments. YM and TB conducted *in vitro* T cell experiments. HS, MR, JH, MN, WA, CD, GI, and JW provided new reagents/analytic tools/samples. HS, MR, JH, MN, WA, CD, GI, and JW collected patient data. AN, YM, and JW analyzed data. AN, YM, and JW wrote the manuscript. All authors revised the manuscript. H-GR and JW supervised the study. All authors contributed to the article and approved the submitted version.

## Funding

This work was supported by the Deutsche Forschungsgemeinschaft (DFG, German Research Foundation, Grant WA 4608/1-2), the Deutsche Forschungsgemeinschaft under Germany’s Excellence Strategy (Grant EXC2180-390900677), the German Cancer Consortium (DKTK), the Wilhelm Sander Stiftung (Grant 2016.177.2), the José Carreras Leukämie-Stiftung (Grant DJCLS 05 R/2017), and the Fortüne Program of the University of Tübingen (Fortüne number 2451-0-0 and 2581-0-0).

## Conflict of Interest

H-GR is shareholder of Immatics Biotechnologies GmbH, Synimmune GmbH, and Curevac AG, and holds a patent application on an adjuvant, XS15. AN, H-GR, and JW are listed as inventors on patents related to peptides described in this manuscript.

The remaining authors declare that the research was conducted in the absence of any commercial or financial relationships that could be construed as a potential conflict of interest.

## Supplementary Material

The Supplementary Information contains the **Supplementary Figures 1–5** and the **Supplementary Tables 1–8**.
