# Supplementary material for: Immunopeptidomics-Guided Warehouse Design for Peptide-Based Immunotherapy in Chronic Lymphocytic Leukemia
Source: Front Immunol. 2021 Jul 8;12:705974. doi: 10.3389/fimmu.2021.705974 (PMC8297687; doi:10.3389/fimmu.2021.705974)
Supplement: Supplementary file 1 [file DataSheet_1.pdf]

Supplementary Material

1 Supplementary Figures

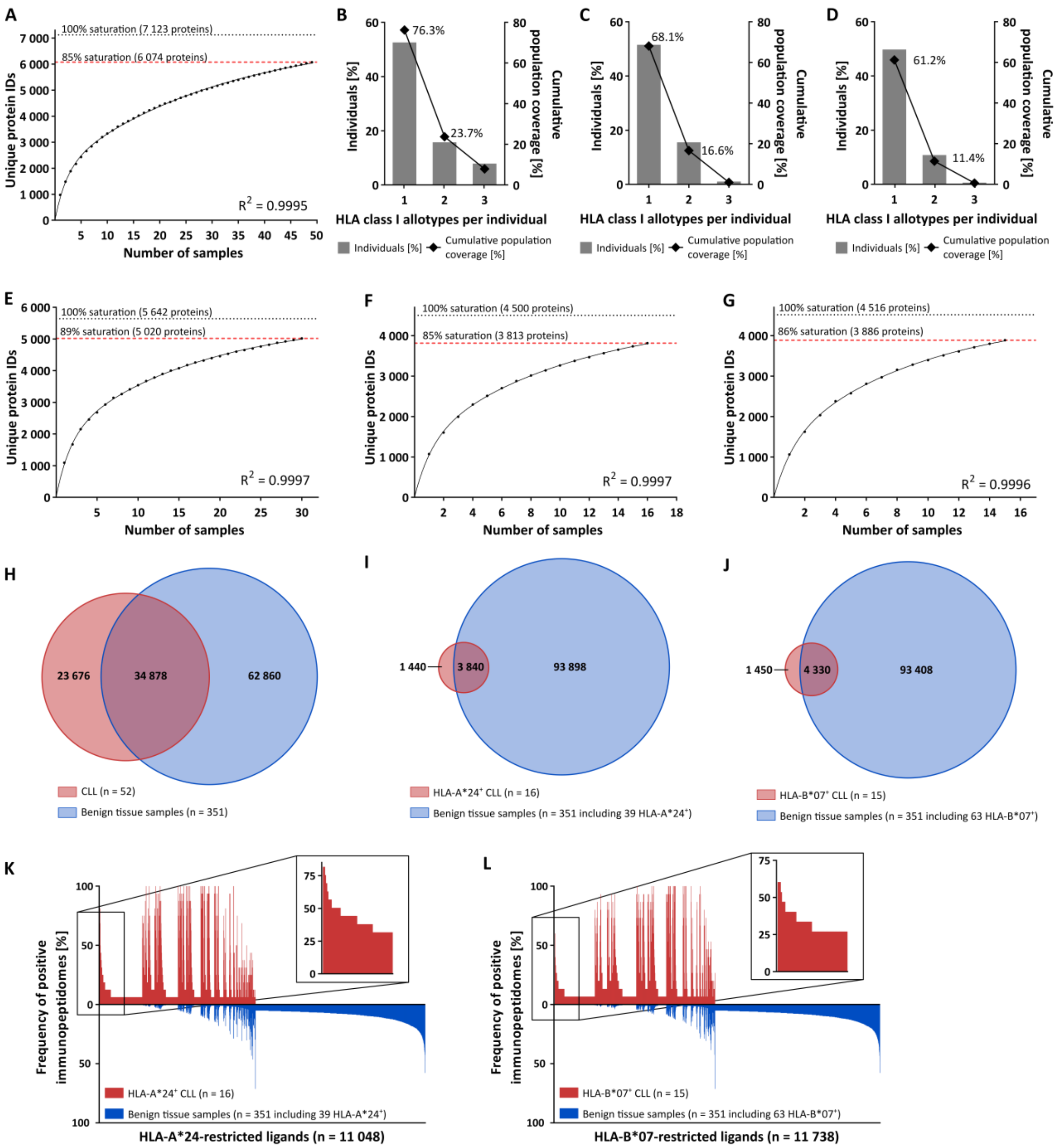

Supplementary Figure 1: Saturation analysis, population coverage and comparative immunopeptidome profiling. (A) Saturation analysis of HLA class II-restricted peptide source proteins of the CLL patient cohort. Number of unique source protein identifications shown as a function of cumulative immunopeptidome analysis of CLL samples (n = 49). Exponential

regression allowed for the robust calculation of the maximum attainable number of different source protein identifications (dotted lines). The dashed red line depicts the source proteome coverage achieved in the CLL patient cohort. **(B-D)** HLA-A*02, HLA-A*24, and HLA-B*07 allotype coverage within **(B)** the CLL patient cohort of a previous peptide vaccination trial (NCT02802943) as well as within **(C)** the European population and **(D)** the world population (calculated by the IEDB population coverage tool, www.iedb.org). The frequencies of individuals within the respective cohort carrying up to three HLA allotypes (x-axis) are indicated as gray bars on the left y-axis. The cumulative percentage of population coverage is depicted as black dots on the right y-axis. **(E-G)** Saturation analysis of **(E)** HLA-A*02-, **(F)** HLA-A*24-, and **(G)** HLA-B*07-restricted peptide source proteins of the CLL patient cohort. Number of unique source protein identifications shown as a function of cumulative immunopeptidome analysis of CLL samples (E, n = 30; F, n = 16; G, n = 15). Exponential regression allowed for the robust calculation of the maximum attainable number of different source protein identifications (dotted lines). The dashed red lines depict the source proteome coverage achieved in the respective CLL patient cohort. **(H-J)** Overlap analysis of **(H)** HLA class I-, **(I)** HLA-A*24-, and **(J)** HLA-B*07-restricted peptides of HLA-matched CLL samples (H, n = 52; I, n = 16; J, n = 15) and benign tissue samples (n = 351; I, including 39 HLA-A*24^{+}; J, including 63 HLA-B*07^{+}). **(K, L)** Comparative profiling of **(K)** HLA-A*24- and **(L)** HLA-B*07-presented ligands based on the frequency of presentation in allotype-matched CLL and benign tissue immunopeptidomes. Frequencies of positive immunopeptidomes for the respective HLA ligands (x-axis) are indicated on the y-axis. HLA ligands identified on < 5% of the respective cohort were not depicted. Boxes on the left side highlight CLL-associated antigens that show CLL-exclusive high-frequent presentation. Abbreviation: IDs, identifications.

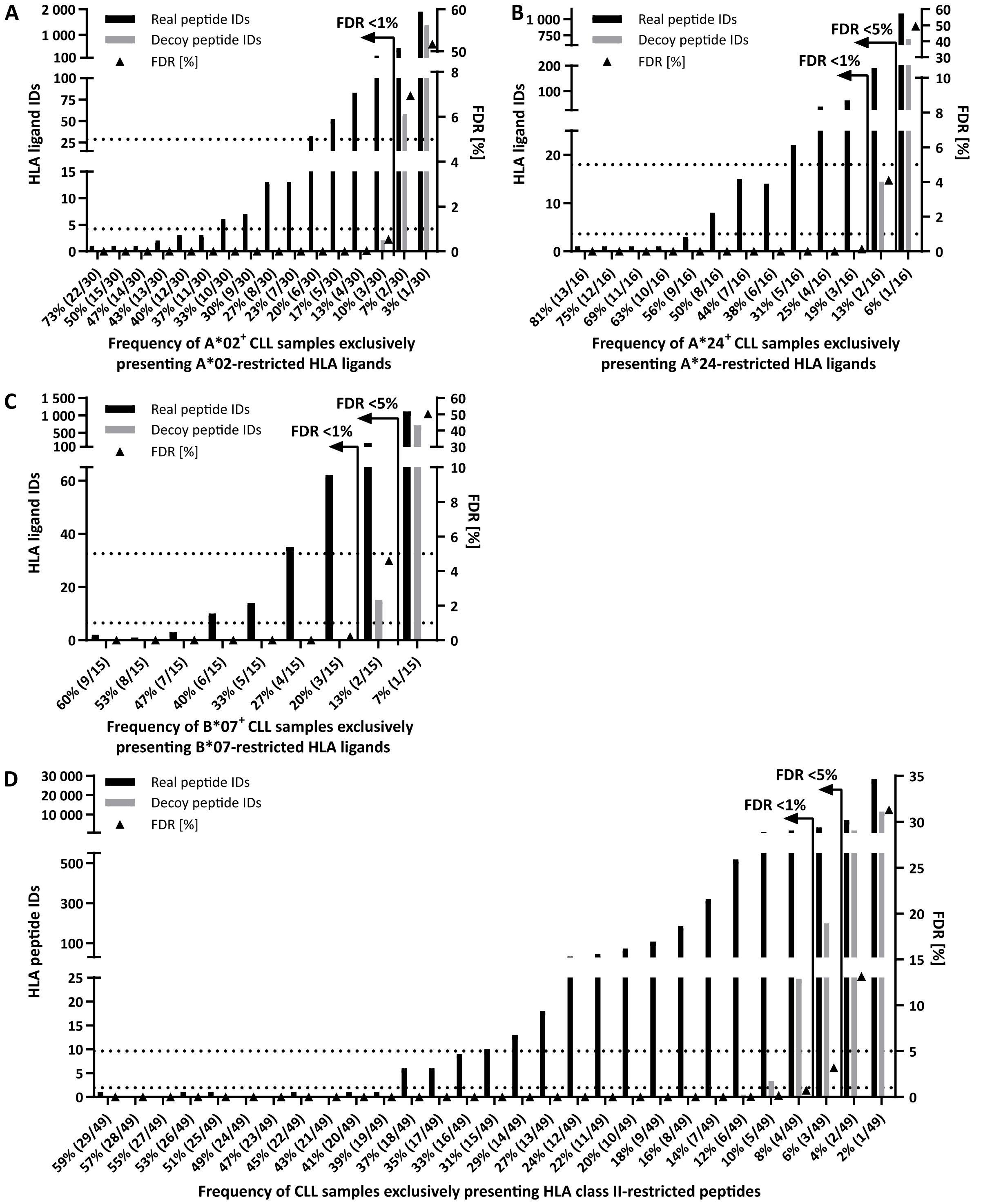

Supplementary Figure 2: Statistical analysis of the proportion of false-positive CLL-associated antigen identifications at different representation frequencies. The numbers of identified **(A)** HLA-A*02-, **(B)** HLA-A*24-, **(C)** HLA-B*07-, and **(D)** HLA class II-restricted peptides based on the analysis of the CLL and benign tissue cohorts were compared with random virtual (HLA-matched) CLL-associated peptides (left y-axis), respectively. Virtual ligandomes of

CLL samples and benign tissue samples were generated *in silico* based on random weighted sampling from the entirety of peptide identifications in both original cohorts. These randomized virtual ligandomes were used to define CLL-associated antigens based on simulated cohorts of CLL *versus* benign tissue samples. The process of peptide randomization, cohort assembly, and CLL-associated antigen identification was repeated 1 000 times and the mean value of resultant virtual CLL-associated antigens was calculated and plotted for the different threshold values. The corresponding false discovery rates (right y-axis) for any chosen threshold (x-axis) were calculated and the 1% and 5% false discovery rates are indicated within the plot (dotted lines and arrows). Abbreviations: IDs, identifications; FDR, false discovery rate.

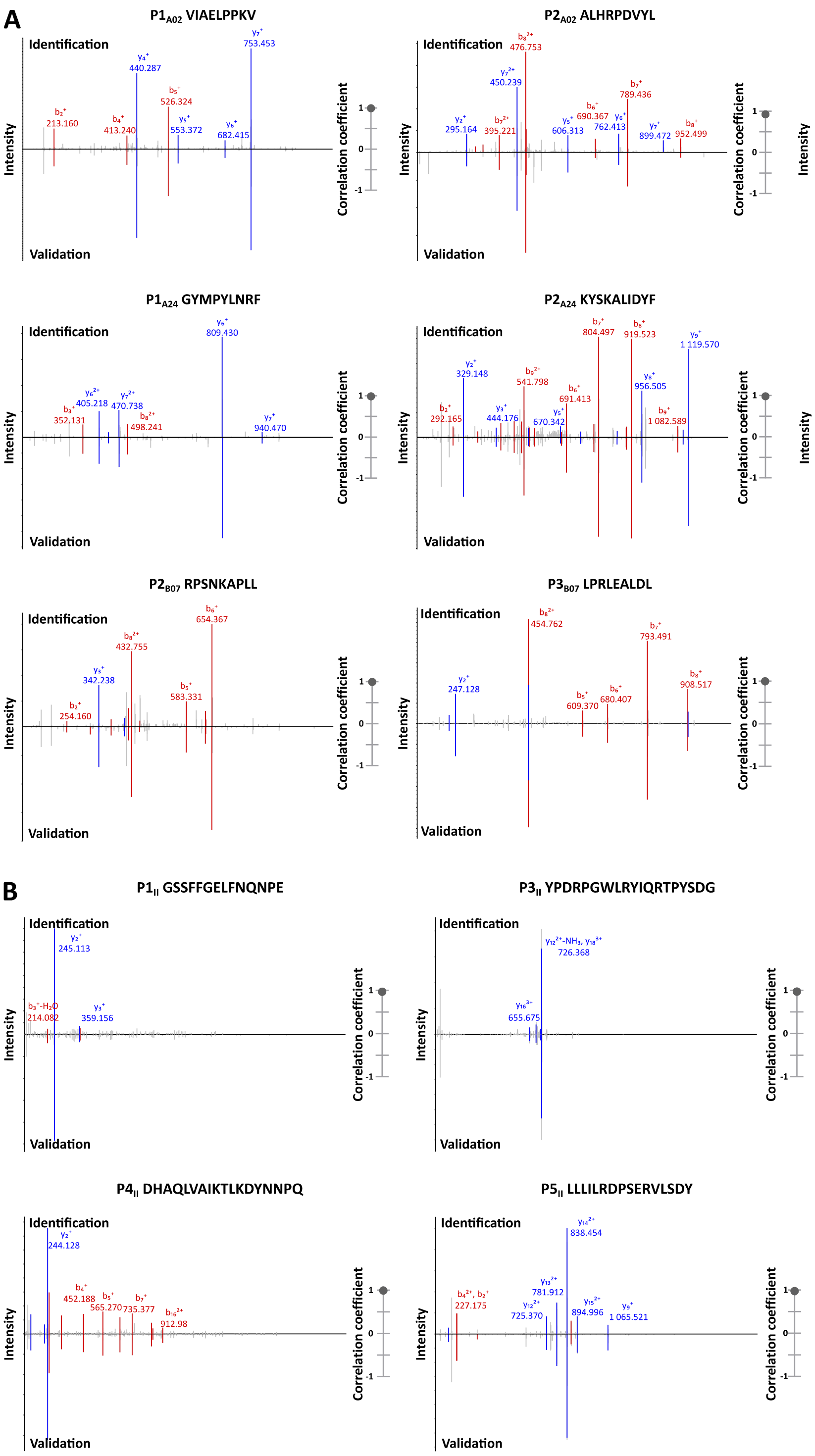

Supplementary Figure 3: Validation of experimentally eluted peptides by synthetic peptides. Comparison of fragment spectra (m/z on the x-axis) of **(A)** HLA class I and **(B)** class II-restricted peptides eluted from primary CLL samples (identification) to their corresponding synthetic peptides (validation, mirrored on x-axis). Identified b- and y-ions are marked in red and blue, respectively. The calculated spectral correlation coefficients are depicted on the right graphs.

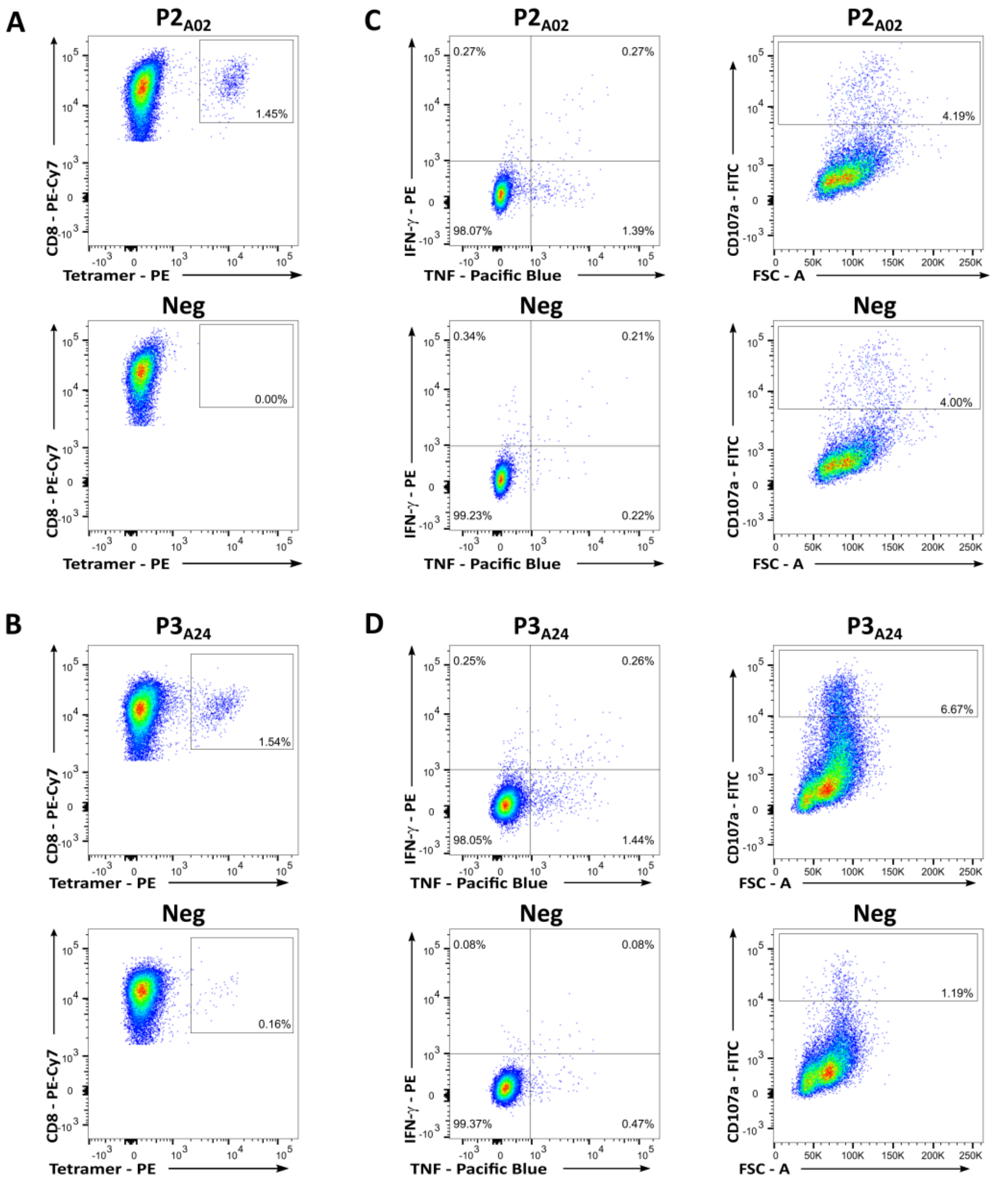

Supplementary Figure 4: Immunogenicity confirmation of HLA-A*02- and HLA-A*24-restricted peptides by *de novo* T cell induction in HVs. (A, B) Tetramer staining of $CD8^+$ T cells derived from HVs after 4 cycles of aAPC stimulation with the peptides **(A)** $P2_{A02}$ and **(B)** $P3_{A24}$. **(C, D)** Representative intracellular cytokine (IFN-γ, TNF) and degranulation marker (CD107a) staining of peptide-specific $CD8^+$ T cells after 4 cycles of *in vitro* aAPC-based primings following stimulation with the peptides **(C)** $P2_{A02}$ and **(D)** $P3_{A24}$. Abbreviations: Neg, negative control; FSC, forward scatter.

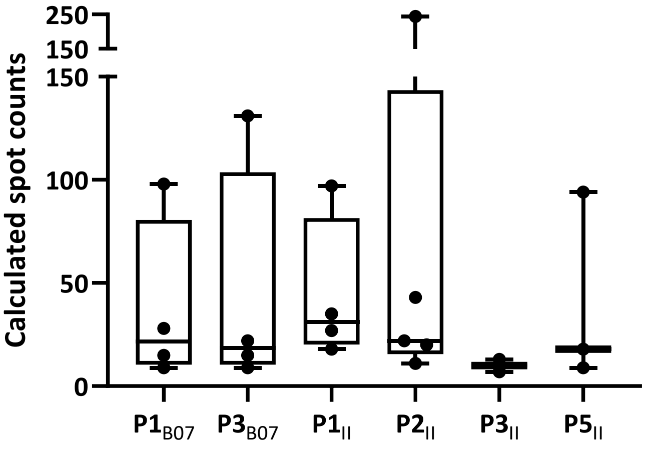

Supplementary Figure 5: Intensity of preexisting T cell responses. Overview of all detected preexisting T cell responses against the respective CLL-associated antigens evaluated by IFN-γ ELISpot assays after 12-day *in vitro* expansion using peripheral blood mononuclear cells (PBMCs) of CLL patients. Intensity is depicted as calculated spot counts, which were calculated as the mean spot count of duplicates normalized to 5×10^5 cells minus the normalized mean spot count of the respective negative control. Each dot represents an individual donor. Boxes represent median and 25th to 75th percentiles, whiskers are minimum to maximum.

2 Supplementary Tables

Supplementary Table 1: Patient characteristics of immunopeptidome samples

UPN	HLA type	Time point sample collection	Therapy prior to sample collection (1st line | 2nd line |…)	WBC [per µl]	Lympho. [%]
**UPN001**	A*02:01, A*11:01, B*39:01, B*40:01, C*03:04, C*12:03	diagnosis	no	245 200	71
**UPN002**	A*02:01, B*35:01, B*39:01, C*04:01, C*12:03	relapse	yes (FC | R-Benda | FC | R-Benda | CLB)	151 690	n.a.
**UPN003**	A*25:01, A*26:01, B*18:01, B*38:01, C*12:03	relapse	yes (CLB)	53 020	56
**UPN004**	A*01:01, A*24:02, B*08:01, B*27:05, C*02:02, C*07:02	diagnosis	no	103 000	75
**UPN005**	A*02:01, A*03:01, B*18:01, B*35:01, C*04:01, C*05:01	diagnosis	no	103 800	77
**UPN006**	A*03:01, A*30:01, B*07:02, B*13:02, C*06:02, C*07:02	relapse	yes (CLB)	130 450	97
**UPN007**	A*02:01, A*03:01, B*07:02, B*55:01, C*01:02, C*07:02	relapse	yes (Trofosfamide)	72 080	84
**UPN008**	A*01:01, A*02:01, B*27:05, B*37:01, C*02:02, C*06:02	diagnosis	no	111 000	86
**UPN009**	A*01:01, A*02:01, B*27:02, B*37:01, C*02:02, C*06:02	diagnosis	no	111 800	79
**UPN010**	A*23:01, B*49:01, C*03:04, C*07:02	relapse	yes (R-Benda | Benda)	37 800	88
**UPN011**	A*01:01, A*68:01, B*08:01, B*44:02, C*07:01, C*07:04	diagnosis	no	40 990	59
**UPN012**	A*02:01, A*03:01, B*40:01, C*03:04	diagnosis	no	35 400	86
**UPN013**	A*01:01, A*03:01, B*08:01, B*51:01, C*01:02, C*07:01	diagnosis	no	54 360	84
**UPN014**	A*02:01, A*03:01, B*07:02, B*44:02, C*05:01, C*07:02	diagnosis	no	61 760	64
**UPN015**	A*02:05, A*24:02, B*35:01, B*50:01, C*04:01, C*06:02	relapse	yes (“Knospe protocol” | R-Benda | Benda | FC | CLB | Benda)	56 360	67
**UPN016**	A*11:01, B*15:01, B*52:01, C*04:01, C*12:02	relapse	yes (R-Benda)	83 390	81
**UPN017**	A*01:01, A*02:01, B*08:01, B*13:02, C*06:02, C*07:02	diagnosis	no	67 410	78
**UPN018**	A*32:01, A*68:01, B*07:02, B*27:05, C*07:01, C*07:02	relapse	yes (R-Benda)	76 840	88
**UPN019**	A*01:01, A*26:01, B*07:02, B*37:01, C*02:02, C*07:02	diagnosis	no	220 710	n.a.
**UPN020**	A*01:01, A*32:01, B*07:02, B*44:02, C*07:01, C*07:02	relapse	yes (R-Benda | CLB)	770 400	n.a.
**UPN021**	A*02:01, A*24:02, B*51:01, B*57:01, C*14:02	relapse	yes (Fludarabine | R-Benda)	110 300	71
**UPN022**	A*02:01, A*03:01, B*40:01, B*44:02, C*02:02, C*03:04	diagnosis	no	46 170	80
**UPN023**	A*02:01, A*03:01, B*35:01, C*04:01	diagnosis	no	160 600	77
**UPN024**	A*01:01, A*02:01, B*08:01, B*51:01, C*02:02, C*07:02	diagnosis	no	123 700	85
**UPN025**	A*02:01, A*24:02, B*51:01, B*57:01, C*01:02, C*06:02	relapse	yes (CLB)	259 750	95
**UPN026**	A*01:01, A*03:01, B*07:02, B*44:02, C*05:01, C*07:02	diagnosis	no	171 510	90
**UPN027**	A*01:01, A*02:01, B*08:01, B*27:02, C*02:02, C*07:01	diagnosis	no	335 730	94
**UPN028**	A*02:01, B*15:01, B*56:01, C*01:02, C*03:04	relapse	yes (R-Benda)	58 260	87
**UPN029**	A*24:02, A*26:01, B*27:05, B*39:01, C*01:02, C*07:02	diagnosis	no	68 610	86
**UPN030**	A*02:01, B*07:02, B*18:01, C*03:04, C*06:02	diagnosis	no	31 950	n.a.
**UPN031**	A*02:01, A*11:01, B*35:01, B*40:01, C*03:04, C*04:01	diagnosis	no	537 370	96
**UPN032**	A*01:01, A*24:02, B*07:02, B*08:01, C*07:02	diagnosis	no	146 810	92
**UPN033**	A*02:01, A*68:01, B*38:01, B*51:01, C*12:03, C*14:02	diagnosis	no	38 680	88
**UPN034**	A*02:01, A*29:02, B*44:02, C*05:01, C*16:01	diagnosis	no	170 420	97
**UPN035**	A*01:01, A*26:01, B*07:02, B*40:01, C*02:02, C*07:02	diagnosis	no	81 510	87
**UPN036**	A*02:01, A*11:01, B*35:01, B*40:01, C*03:04, C*04:01	diagnosis	no	27 340	73
**UPN037**	A*02:01, A*24:02, B*15:01, B*44:02, C*03:04, C*16:01	diagnosis	no	438 060	97
**UPN038**	A*24:02, A*25:01, B*18:01, B*49:01, C*07:02, C*12:03	diagnosis	no	227 910	96

UPN	HLA type	Time point sample collection	Therapy prior to sample collection (1st line | 2nd line |…)	WBC [per µl]	Lympho. [%]
**UPN039**	A*03:01, A*24:02, B*35:01, C*04:01	diagnosis	no	79 010	92
**UPN040**	A*02:01, A*24:02, B*07:02, B*44:02, C*05:01, C*07:02	diagnosis	no	147 340	92
**UPN041**	A*02:01, A*11:01, B*07:02, B*15:01, C*03:04, C*07:02	diagnosis	no	127 100	94
**UPN042**	A*02:01, A*24:02, B*07:02, B*13:02, C*06:02, C*07:02	diagnosis	no	259 550	94
**UPN043**	A*01:01, A*68:01, B*44:02, B*55:01, C*03:04, C*05:01	diagnosis	no	253 100	97
**UPN044**	A*01:01, A*24:02, B*08:01, B*35:01, C*04:01, C*07:02	diagnosis	no	129 080	99
**UPN045**	A*03:01, A*24:02, B*51:01, C*14:02, C*16:01	diagnosis	no	198 200	97
**UPN046**	A*02:01, A*24:02, B*13:02, B*44:02, C*05:01, C*06:02	diagnosis	no	401 080	91
**UPN047**	A*02:01, A*26:01, B*07:02, B*40:01, C*03:04, C*07:02	diagnosis	no	115 000	63
**UPN048**	A*02:01, A*11:01, B*40:01, B*44:02, C*03:04, C*05:01	diagnosis	no	79 800	35
**UPN049**	A*02:01, B*08:01, B*40:01, C*03:04, C*07:02	diagnosis	no	95 200	55
**UPN050**	A*11:01, A*68:01, B*08:01, B*35:01, C*04:01, C*07:02	diagnosis	no	166 000	59
**UPN051**	A*01:01, A*24:02, B*15:01, B*40:01, C*02:02, C*07:02	diagnosis	no	123 500	78
**UPN052**	A*01:01, A*24:02, B*07:02, B*08:01, C*07:01, C*07:02	diagnosis	no	51 850	91
**UPN053**	A*02:01, A*30:01, B*13:02, B*35:01	diagnosis	no	52 350	79
**UPN054**	A*02:01, B*40:01, B*51:01, C*03:04, C*15:02	diagnosis	no	238 570	94
**UPN055**	A*02:01, B*55:01, B*57:01	relapse	yes (R-CHOP*)	83 560	n.a.
**UPN056**	A*30:01, A*33:01, B*14:02, B*40:01, C*03:04, C*08:02	diagnosis	no	20 000	61
**UPN057**	A*02:01, A*03:01, B*27:05, B*57:01	diagnosis	no	43 000	78
**UPN058**	A*02:01, A*24:02, B*07:02, B*51:01, C*01:02, C*07:02	diagnosis	no	27 300	90
**UPN059**	A*03:01, A*26:01, B*07:02, B*38:01, C*07:02, C*12:03	diagnosis	no	36 870	89
**UPN060**	A*01:01, A*02:01, B*51:01, B*57:01, C*06:02, C*15:02	diagnosis	no	20 300	69
**UPN061**	A*02:01, A*11:01, B*39:06, C*06:02	diagnosis	no	131 400	98

UPN, uniform patient number; WBC, white blood cell count; lympho., lymphocytes; n.a., not available; Benda, Bendamustine; FC, Fludarabine and Cyclophosphamide; R-CHOP, Rituximab - Cyclophosphamide - Hydroxydaunorubicin - Oncovin - Prednisone; R-Benda, Rituximab - Bendamustine; CLB, Chlorambucil; "Knospe protocol", Chlorambucil and Prednisone; *, individualized therapy concept due to high risk disease (TP53 mutation).

Supplementary Table 2: Sample characteristics of immunogenicity cohort

UPN	Lymphocyte count [per µl]	$CD3^{+}$ cells [% of lymphocytes]
**UPN005**	147 970	2.7
**UPN007**	1 340	71.3
**UPN012**	11 580	n.a
**UPN017**	148 270	3.9
**UPN018**	67 680	n.a
**UPN019**	99 140	2.6
**UPN022**	14 820	n.a
**UPN026**	202 240	2.6
**UPN029**	59 040	3.6
**UPN035**	71 020	7.7
**UPN038**	219 200	2.4
**UPN039**	72 730	4.2
**UPN040**	136 130	1.8
**UPN041**	119 420	7.2
**UPN042**	243 070	4.0
**UPN045**	192 360	n.a
**UPN047**	72 200	n.a
**UPN051**	77 810	n.a
**UPN052**	47 410	n.a
**UPN057**	61 570	3.7
**UPN058**	24 530	6.3
**UPN062**	730	n.a
**UPN063**	28 890	n.a
**UPN064**	1 830	n.a
**UPN066**	66 010	n.a
**UPN069**	55 530	n.a
**UPN070**	3 720	44.5
**UPN071**	2 850	n.a
**UPN073**	38 400	7.9
**UPN074**	31 569	n.a
**UPN075**	106 370	n.a
**UPN077**	68 940	7.9
**UPN078**	172 680	1.8
**UPN080**	580	n.a
**UPN082**	1 660	n.a
**UPN083**	100 260	6.1
**UPN085**	153 520	2.5
**UPN087**	19 850	4.9
**UPN088**	176 120	n.a
**UPN089**	193 260	n.a
**UPN090**	136 130	1.8
**UPN091**	129 840	3.7
**UPN092**	510	36.0
**UPN093**	121 150	n.a
**UPN095**	34 650	4.2
**UPN096**	329 310	1.6
**UPN097**	13 550	12.2
**UPN098**	17 370	99.2
**UPN099**	5 630	31.8
**UPN100**	6 430	9.8
**UPN102**	2 100	70.7

Supplementary Table 3: Recurrent CLL-associated mutations

Gene	Protein	Mutation [protein sequence]
**Frameshift mutations**		
BIRC3	BIRC3	Q547Nfs*21
NFKBIE	IKBE	Y254Sfs*13
NOTCH1	NOTC1	P2514fsRVP*
**Missense mutations**		
**Gene**	**Protein**	**Mutation [protein sequence]**
ADGRF5	ADGRF5	S765F
ASB10	ASB10	H227L
ATM	ATM	L1222P, R3008C
BIRC6	BIRC6	L3687R
BNC2	BNC2	V771I
BRAF	BRAF	G469A, V600E
CALCRL	CARL1	Q33H
CARMIL1	CARMIL1	L283H
CCDC185	CC185	R380W
CCND3	CCND3	P199S
CD3G	CD3G	D35G
CEP250	CP250	Q2041H
COL3A1	CO3A1	G741S
DNASE2	DNS2A	R62W
EDEM2	EDEM2	V39F
EGR2	EGR2	E356K, H384N
EIF2A	EIF2A	D359N
ERBB4	ERBB4	R50H
GLB1L2	GLBL2	G366S
HOXC11	HXC11	E202D
IKZF3	IKZF3	L162R
IRF4	IRF4	S114R
JAK2	JAK2	V617F
KCTD9	KCTD9	P199S
KIAA1211	CRAD	A1035V
KIAA1217	SKT	R1188W
LGI3	LGI3	W341R
LRP1B	LRP1B	N1754K
LRRD1	LRRD1	P735S
LRRIQ4	LRIQ4	V225I
MDGA2	MDGA2	R53W
MED12	MED12	E33K, L36R, G44D, G44S
MYO5B	MYO5B	T846I
NFE2L3	NF2L3	C595Y
NID1	NID1	G562S
NRAS	RASN	Q61K, Q61R
PADI3	PADI3	T335I
PAPPA	PAPPA	G438R
PCDHA13	PCDAD	D376Y
PENK	PENK	R229W
PLCG2	PLCG2	S707F, D993H, M1141R, M1141K
RIMS1	RIMS1	E658A
RYR3	RYR3	D4710N
SBNO1	SBNO1	V1132A
SECISBP2	SEBP2	K15R
SF3B1	SF3B1	E622D, Y623C, N626Y, H662D, H662Q, T663I, K666E, K666N, K666T, K700E, I704F, I704N, G740E, G742D
SLC7A13	S7A13	V407M
SNX16	SNX16	W171L
STAMBPL1	STALP	P48R
TLR2	TLR2	D327V

Gene	Protein	Mutation [protein sequence]
TMEM131	T131L	S1256C
TP53	P53	R175H, H179L, Y220C, Y234C, S241F, G244D, R248Q, R248W, R273C, R273H, R337C
TRAPPC10	TPC10	A301S
TSPAN12	TSN12	E301K
UGT2B4	UD2B4	I331V
UTF1	UTF1	R108L
VPS13D	VP13D	A4248V
XPO1	XPO1	E571A, E571G, E571K, E571Q, E571V

Supplementary Table 4: HLA class I and HLA class II peptide yields

UPN	Mass spec	HLA class I		HLA-A*02		HLA-A*24		HLA-B*07		HLA class II	
		Ligand IDs	Protein IDs	Ligand IDs	Protein IDs	Ligand IDs	Protein IDs	Ligand IDs	Protein IDs	Peptide IDs	Protein IDs
**UPN001**	XL	1 554	1 432	201	265					997	327
**UPN002**	XL	852	929	251	358					706	473
**UPN003**	XL	1 230	1 199							n.a.	n.a.
**UPN004**	XL	1 921	1 800			509	614			1 075	470
**UPN005**	XL	1 349	1 375	201	280					575	363
**UPN006**	XL	1 407	1 563					486	557	n.a.	n.a.
**UPN007**	XL	2 555	2 196	919	1 016			1 263	1 212	n.a.	n.a.
**UPN008**	XL	1 121	1 198	299	387					n.a.	n.a.
**UPN009**	XL	1 492	1 457	666	736					944	441
**UPN010**	XL	679	771							n.a.	n.a.
**UPN011**	XL	1 228	1 203							n.a.	n.a.
**UPN012**	XL	1 157	1 173	247	336					n.a.	n.a.
**UPN013**	XL	1 243	1 288							627	331
**UPN014**	XL	1 182	1 206	177	250			466	500	n.a.	n.a.
**UPN015**	XL	632	759	174	256	199	303			n.a.	n.a.
**UPN016**	XL	800	849							n.a.	n.a.
**UPN017**	XL	1 060	1 130	533	665					691	326
**UPN018**	XL	678	729					223	257	631	360
**UPN019**	XL	1 308	1 247					794	783	n.a.	n.a.
**UPN020**	XL	759	786					239	266	1 651	643
**UPN021**	XL	728	784	159	220	319	391			1 066	551
**UPN022**	XL	2 457	1 992	511	632					728	379
**UPN023**	XL	1 231	1 221	266	364					n.a.	n.a.
**UPN024**	XL	849	969	82	93					725	388
**UPN025**	XL	527	676	99	177	134	201			745	405
**UPN026**	XL	1 860	1 714					658	692	3 029	877
**UPN027**	Lumos	8 561	4 741	2 811	2 312					7 865	1 393
**UPN028**	Lumos	7 957	4 912	3 723	2 845					7 010	1 406
**UPN029**	Lumos	3 082	2 599			1 505	1 499			4 096	664
**UPN030**	Lumos	5 976	3 820	2 500	2 125			1 815	1 642	2 586	815
**UPN031**	Lumos	8 622	4 610	2 198	1 936					7 397	1 430
**UPN032**	Lumos	4 928	3 570			1 679	1 636	1 990	1 781	6 488	1 411
**UPN033**	Lumos	5 298	3 568	1 594	1 542					6 748	1 411
**UPN034**	Lumos	9 530	5 046	2 134	1 865					9 570	1 789
**UPN035**	Lumos	3 055	2 436					651	720	2 603	842
**UPN036**	Lumos	2 003	1 725	456	574					3 316	928
**UPN037**	Lumos	3 412	2 593	731	853	973	1 040			3 470	896
**UPN038**	Lumos	1 992	1 822			533	639			8 922	1 649
**UPN039**	Lumos	4 393	3 285			1 515	1 550			8 954	1 799
**UPN040**	Lumos	8 816	4 917	2 182	1 938	1 926	1 866	2 933	2 361	10 392	1 626
**UPN041**	Lumos	7 539	4 644	1 908	1 749			2 568	2 166	7 016	1 531

UPN	Mass spec	HLA class I		HLA-A*02		HLA-A*24		HLA-B*07		HLA class II	
		Ligand IDs	Protein IDs	Ligand IDs	Protein IDs	Ligand IDs	Protein IDs	Ligand IDs	Protein IDs	Peptide IDs	Protein IDs
**UPN042**	Lumos	4 558	3 296	2 042	1 850	1 447	1 439	1 235	1 197	4 272	1 104
**UPN043**	Lumos	2 696	2 284							4 524	999
**UPN044**	Lumos	3 608	2 943			1 264	1 305			7 258	1 558
**UPN045**	Lumos	1 186	1 247			536	609			1 264	509
**UPN046**	Lumos	7 643	4 461	2 969	2 439	1 775	1 742			7 031	1 491
**UPN047**	Lumos	2 490	2 135	927	1 032			508	588	2 569	832
**UPN048**	Lumos	3 002	2 347	1 077	1 131					4 246	1 039
**UPN049**	Lumos	2 877	2 316	1 599	1 544					4 490	1 196
**UPN050**	Lumos	4 358	3 238							6 192	1 551
**UPN051**	Lumos	5 292	3 554			1 439	1 405			5 673	1 337
**UPN052**	Lumos	3 073	2 598			916	978	1 267	1 236	2 624	870
**UPN053**	XL	n.a.	n.a.							606	357
**UPN054**	XL	n.a.	n.a.							873	474
**UPN055**	XL	n.a.	n.a.							935	489
**UPN056**	Lumos	n.a.	n.a.							603	306
**UPN057**	Lumos	n.a.	n.a.							4 505	1 280
**UPN058**	Lumos	n.a.	n.a.							4 584	1 286
**UPN059**	Lumos	n.a.	n.a.							5 343	1 530
**UPN060**	Lumos	n.a.	n.a.							6 233	1 697
**UPN061**	Lumos	n.a.	n.a.							3 814	1 103

UPN, uniform patient number; Mass spec, mass spectrometer; ID, identification; n.a., not available.

Supplementary Table 5: HLA-A*02-restricted CLL-associated antigens

Sequence	Source protein	Peptide length	Allotype-specific presentation frequency	Sequence	Source protein	Peptide length	Allotype-specific presentation frequency
**KLLESVASA**	PACER	9	73%	**LLFHGMLLL**	ZDH24	9	23%
**GIIDGSPRL**	PACER	9	50%	**QLYNSLIFL**	RNFT2	9	23%
**VIAELPPKV**	IGHM	9	47%	**SLASITVPL**	GGA1	9	23%
**SLFSHLLEI**	WDFY4	9	43%	**SLLAELHVLTV**	FCRL3	11	23%
**VLTNLVVFL**	ABCA6	9	43%	**SLMLEVPAL**	DMD	9	23%
**ALHRPDVYL**	IGHM	9	40%	**SLPELVHAV**	SESN3	9	23%
**YLLDQSFVM**	RGRF1	9	40%	**SLTSLLILV**	TM243	9	23%
**YLTVVIFTA**	LOX5	9	40%	**VLRELCEEL**	SYNE2	9	23%
**ILDEKPVII**	ABCA6	9	37%	**YILTFPLYL**	MET7A	9	23%
**RLLYQLVFL**	IL4RA	9	37%	**AIPPSFASIFL**	IGHM	11	20%
**TLDTSKLYV**	RGRF1	9	37%	**ALHWFLNQV**	UBP34	9	20%
**FLTDLEDLTL**	NAT9	10	33%	**ALMGLSAQL**	DNMBP	9	20%
**LIWPLLSTV**	NUP88	9	33%	**ALWIPEVSI**	JADE1, JADE2, JADE3	9	20%
**LLDAMNYHL**	KLH14	9	33%	**ATMPVVPSV**	SHLB2	9	20%
**SLASHIQSL**	WDFY4	9	33%	**FAIPPSFASI**	IGHM	10	20%
**VMLQINPKL**	GRDN	9	33%	**FLNFNSFNL**	CC14B, CC14C	9	20%
**YLVEDVLLL**	KLH14	9	33%	**FLYIGDIVSL**	ITPR2	10	20%
**ALPEILFAKV**	CXCR5	10	30%	**FVDEGIKTL**	DPOLB	9	20%
**AVAIIVVSV**	SCIMP	9	30%	**FVFEAPYTL**	DOC11	9	20%
**FLSAMDWHL**	CNPD1	9	30%	**GLLRASFLL**	TLR9	9	20%
**LLHEIENHL**	PKHG1	9	30%	**GLYFGMLLL**	CD37	9	20%
**LLLPDVIKV**	TBCD9	9	30%	**HLANIVERL**	TRI34	9	20%
**VLTDIVAKC**	DOC10	9	30%	**HLIDTNKIQL**	DOC10	10	20%
**YLGGFALSV**	KSYK	9	30%	**IIQSYIINI**	PCDBI	9	20%
**ALGIFSFTL**	SGPP1	9	27%	**ILSLSIASV**	CYAC3	9	20%
**ALPTLIPSV**	ZEP1	9	27%	**KQSEEIPEV**	LONF1	9	20%
**ALVDELEWEI**	CNPY2	10	27%	**KVIGFLEEV**	RGRF1	9	20%
**ALYLTEVFL**	BANK1	9	27%	**NLWSVDGEVTV**	SNX29	11	20%
**AVGAFLIYI**	MAT2B	9	27%	**RVLEALWEL**	BAIP3	9	20%
**FLIDGSFNI**	COCH	9	27%	**SLAHVAGCEL**	PACER	10	20%
**FTLPEVAEC**	HNRPU	9	27%	**SLASIHVPL**	GGA3	9	20%
**GLLDGVFNV**	CRNS1	9	27%	**SLDLTTTCV**	FOXP1	9	20%
**IINGIIISV**	HVCN1	9	27%	**SLMGTVFLL**	SAMD8	9	20%
**QLIPKLIFL**	WDFY4	9	27%	**SLMSVGFLL**	NUBP2	9	20%
**SLFDLDGPKV**	PHF23	10	27%	**SVASVLLYL**	PRKDC	9	20%
**SLFLGILSV**	CD20	9	27%	**SVWEKEIEI**	AKAP9	9	20%
**SLLAELHVL**	FCRL3	9	27%	**VLLSIPFVSV**	ORML3	10	20%
**ATPMPTPSV**	SBNO1	9	23%	**YLFEEAISM**	WDFY4	9	20%
**GLGELAGLTV**	STRN	10	23%	**YLMAAEDLEL**	CK5P2	10	20%
**HVLEEVQQV**	CKAP4	9	23%	**YLVNFLHKL**	TEAD2	9	20%
**KLTEENTTL**	PEG10	9	23%	**YQFDSALLPAV**	SPIB	11	20%

Supplementary Table 6: HLA-A*24-restricted CLL-associated antigens

Sequence	Source protein	Peptide length	Allotype-specific presentation frequency	Sequence	Source protein	Peptide length	Allotype-specific presentation frequency
**TYTDVTPRQF**	STAR7	10	81%	**LYQTFVVQL**	IL2RG	9	31%
**IYQQNHMVL**	IKZF3	9	75%	**LYSQLQVFF**	TRM7	9	31%
**KYGVFEESL**	TRI34	9	69%	**MYPVWKSFL**	FCSD2	9	31%
**VYNENLVHM**	SPF27	9	63%	**PYPQYLAVI**	MED29	9	31%
**GYMPYLNRF**	SWP70	9	56%	**QYILIHQAL**	PTPRC	9	31%
**KYVGAVQML**	SNX29	9	56%	**RYVRKFVLM**	CHM2A	9	31%
**RFPPTPPLF**	BC11A	9	56%	**SYGYQFPGF**	MSI2H	9	31%
**FYVGHIDAF**	ICE2	9	50%	**TYDAHHSAF**	IRF8	9	31%
**IFPPVINITW**	DOA	10	50%	**TYIKVFVPSW**	ZC12D	10	31%
**IFPPVVNITW**	DQA2	10	50%	**VFKLWPLSF**	PIGQ	9	31%
**KYSKALIDYF**	AFF3	10	50%	**VWSDIAPLNF**	MMP17	10	31%
**KYTEGVQSL**	SP16H	9	50%	**VYPTLSQQL**	TPC10	9	31%
**RHTGALPLF**	SI1L3	9	50%	**YFISHILAF**	RIR2B	9	31%
**VHIPEVYLI**	WDFY4	9	50%	**AYPTAYPSF**	SMAP2	9	25%
**VYHSDIPKW**	SIAT1	9	50%	**DYLEWPEYF**	DCTD	9	25%
**AFPEIFYTF**	PI3R4	9	44%	**EFKQFAQLF**	TRAF5	9	25%
**EYSRFVNQI**	KHDC4	9	44%	**EWPKHWPTF**	XPO1	9	25%
**FYIENMQYL**	ABCA6	9	44%	**EYGENFPML**	ZN121	9	25%
**FYTLIPHDF**	PARP1	9	44%	**FYTQLLQEL**	SMG7	9	25%
**GYPGRQYYF**	RHBL4	9	44%	**GYPVPPYAFF**	RARA	10	25%
**IFLTKSTKL**	IGHM	9	44%	**HYFNTPFQL**	PPTC7	9	25%
**IYGKDVFEAF**	CUL4B	10	44%	**IFNGFSVTL**	MPCP	9	25%
**IYNGETLVF**	PCYOX	9	44%	**IYGSVPYLL**	GANC	9	25%
**IYSPDHTNNSF**	ITF2	11	44%	**IYNHITTRV**	ADDA	9	25%
**IYWDGPLAL**	IRF4	9	44%	**IYQKPFQTL**	ATLA2	9	25%
**KLPTEWNVL**	AKP13	9	44%	**KYAATSQVL**	IGHM	9	25%
**SYLPRIVLL**	GRP3	9	44%	**KYIEYYLVL**	ADA28	9	25%
**TYKALNTFI**	CLPT1	9	44%	**KYLSDNVHL**	CDC37	9	25%
**VFSNVSIILF**	GNA13	10	44%	**KYSFLPYQL**	BFAR	9	25%
**YFYLFPNRL**	ARBK1	9	44%	**KYVKVFDKF**	ZN107	9	25%
**DWPLTQVTF**	FCRLA	9	38%	**LYQHAVEYF**	VPS4A	9	25%
**DYTGALAVF**	HAP40	9	38%	**LYVPALSALW**	GNA12	10	25%
**EYTRYLFAL**	FARP2	9	38%	**NWGRLVAFF**	B2CL2, BCL2	9	25%
**IFTDIFHYL**	XRN1	9	38%	**NYTDRIQVL**	PDE4B	9	25%
**IYSQLETLI**	S11IP	9	38%	**QYVVDLTSF**	NTPCR	9	25%
**KYPASTVQI**	NOP56	9	38%	**RYKEENNDHL**	UBP8	10	25%
**LFKNDPLFF**	LACTB	9	38%	**SEYADTHYF**	CLNK	9	25%
**PYAKPIPAQF**	WDR33	10	38%	**SYILDTLVF**	TNPO2	9	25%
**RYGLPAAWSTF**	IGFR1	11	38%	**TFTDHVMLF**	DDX27	9	25%
**RYNGGLLEF**	DZIP3	9	38%	**TYSEDTYRL**	NR2C2	9	25%
**RYPLLLMEL**	DNMBP	9	38%	**TYSSSYEQF**	SRPK2	9	25%
**TYDSVTISW**	IGHM	9	38%	**TYVKEIEVW**	RN213	9	25%
**VFIIVPAIF**	GPAT4	9	38%	**VAAGSYQRF**	PMF1	9	25%
**VYRQDCETF**	PPHLN	9	38%	**VFIEGADAETF**	SYEP	11	25%
**AYVVFVTTL**	HTR5B	9	31%	**VFTPYSAAFLL**	KCNH2, KCNH6	11	25%
**EFLTKTAKF**	P4R3A, P4R3B	9	31%	**VYERAVEFF**	CRNL1	9	25%
**IYGGTYML**	GDIA, GDIB	8	31%	**VYNIPVRF**	BLNK	8	25%
**IYHFNSELL**	PKHG1	9	31%	**VYPYKLYRL**	ZBT38	9	25%
**IYKDLPFETL**	RM39	10	31%	**VYQVGGVTAYF**	MFRN2	11	25%
**IYVIPQPHF**	KNL1	9	31%	**YLLDQSFVM**	RGRF1	9	25%
**KYDDNVKAYF**	AIP	10	31%	**YWPDVIHSF**	RNT2	9	25%
**LYGKVQEI**	GMDS	8	31%	**YYTVAHAI**	SMCA2	8	25%
**LYPGQLVQL**	RHG09	9	31%				

Supplementary Table 7: HLA-B*07-restricted CLL-associated antigens

Sequence	Source protein	Peptide length	Allotype-specific presentation frequency	Sequence	Source protein	Peptide length	Allotype-specific presentation frequency
**IPSIHIEL**	RHG44	8	60%	**MPLLSRLDL**	KDM2A	9	27%
**SPRSWIQVQI**	FCRL5	10	60%	**NPRYPNYMF**	ROR1	9	27%
**SPRVYWLGL**	CL17A	9	53%	**RPCDISRQL**	PAX1/2/5/8/9	9	27%
**APQHKGHTTAL**	TRI38	11	47%	**RPHVSPRHSF**	RELB	10	27%
**RPKENVTIM**	LY9	9	47%	**SAAHSRQAL**	LFNG	9	27%
**RPSNKAPLL**	EHMT1	9	47%	**SLASHIQSL**	WDFY4	9	27%
**GPGPLRESL**	PRAG1	9	40%	**SPDATRESM**	SNX17	9	27%
**GPMAYARAFL**	DOC10	10	40%	**SPKGRFVML**	FBX7	9	27%
**IPASHPVL**	FCRL5	8	40%	**SPRKSSSI**	ACINU	8	27%
**IPRRQEHDISL**	SYMPK	11	40%	**SPSGNHQSSF**	BMI1	10	27%
**IPVSHPVL**	FCRL3	8	40%	**TIRAIIAVL**	YTDC2	9	27%
**LPRLEALDL**	TLR9	9	40%	**TPKGETRQL**	ABR	9	27%
**RAAENRQGTL**	NCF1	10	40%	**VPSPKVVL**	CPSF2	8	27%
**RPALPRSEL**	M3K14	9	40%	**ALMGLSAQL**	DNMBP	9	20%
**SPGGAHSNL**	ARHGI	9	40%	**APARGLLL**	SPAST	8	20%
**VPRNLPSSL**	TLR9	9	40%	**APEAKKQKV**	NUCL	9	20%
**AAAAGRIAI**	PTBP1	9	33%	**APESKHKSSL**	STT3B	10	20%
**APSFRAGAQL**	DOP2	10	33%	**APLLKDIL**	PACER	8	20%
**APSLQAKL**	TEAD2	8	33%	**APNTGRANQQM**	BFAR	11	20%
**ISRPKGVAL**	IGHM	9	33%	**APRDGRVVF**	SIPA1	9	20%
**KPFSQTPFTL**	TEAD2	10	33%	**APREPFAHSL**	ZC12D	10	20%
**NPSADRNLL**	GGA2	9	33%	**APRGNVTSLSL**	TLR9	11	20%
**QPKGGHVTSM**	KMT2D	10	33%	**ASRKSTAAL**	ARHG7	9	20%
**RAAKETISL**	3BP5	9	33%	**EPAVRSSEL**	SH2D3	9	20%
**RPAVGHSGL**	ZC3H3	9	33%	**EPQPERSSV**	IER5	9	20%
**RPNTTSSTGM**	WIPF2	10	33%	**GPDHNRSFI**	DHX9	9	20%
**RPQKISGNPSL**	FOXP1	11	33%	**GPLVRQISL**	ZEP1	9	20%
**SPFHRNLFL**	WDR34	9	33%	**IPSIRNSILAI**	USP9Y	11	20%
**VPEQRTVTL**	DEPD5	9	33%	**KAKPVTTNL**	RBIS	9	20%
**VPSEPGGVL**	PTN6	9	33%	**KPAENDVKL**	UBCP1	9	20%
**APKPKWTQL**	ALKB6	9	27%	**KPASKKERI**	CHD2	9	20%
**APKPRLNQL**	SYF1	9	27%	**KPDFKELTV**	RINI	9	20%
**APPQIPDTRREL**	EAF6	12	27%	**KPEIRVTSL**	PWP2	9	20%
**APQPAKPRL**	HDAC6	9	27%	**KPGAAMVEM**	HNRPL	9	20%
**APRGNVTSL**	TLR9	9	27%	**KPGAPLQAF**	DEN1C	9	20%
**APRWGNPRAL**	OAS1	10	27%	**KPIEPRRELL**	HSH2D	10	20%
**APSFGSLVAL**	TLR9	10	27%	**KPKPLSQAEM**	AIM2	10	20%
**APTIVGKSSL**	OST48	10	27%	**KPRVTPVEVM**	PAF1	10	20%
**APTPRIKAEL**	TLE1/2/4	10	27%	**KPSEERKTI**	ARI5B	9	20%
**FPKEPVEL**	DPA1	8	27%	**KPYNNHSEM**	DCP2	9	20%
**HPKPSEASTTL**	IFIX	11	27%	**KVKNVGIFL**	SP16H	9	20%
**HPRFLVALI**	SAC2	9	27%	**LPAPSWNVL**	OAS2	9	20%
**IPVSRPIL**	FCRL1	8	27%	**LPRHSFGRNAL**	GBRB2	11	20%
**IYSPDHTNNSF**	ITF2	11	27%	**LPRPQGAAA**	PSRC1	9	20%
**KIRPHIATL**	PUM1	9	27%	**MPSSRAYGL**	NCOA3	9	20%
**KPGKAPKL**	KV133, KV105, KVD33, KV139	8	27%	**NPDWRRLPREL**	RPC7L	11	20%
**KPIGGAAEL**	VP13B	9	27%	**QPEKSKKEL**	NOL9	9	20%
**KPIPLPRF**	BLNK	8	27%	**QPFRDRSNTL**	SYNRG	10	20%
**KPTDEKLREL**	SMC1A	10	27%	**QPSWSIRTAL**	UB2J1	10	20%
**KPYHAHKEEM**	GLYR1	10	27%	**RAAKKKASL**	FOXO1	9	20%
**LPDSDKAIL**	CAR11	9	27%	**RPEDQRSSF**	HPS5	9	20%
**LPSSHVARL**	SMHD1	9	27%	**RPENRAPGAGL**	EMD	11	20%

Sequence	Source protein	Peptide length	Allotype-specific presentation frequency	Sequence	Source protein	Peptide length	Allotype-specific presentation frequency
**RPGAHPLSF**	UHRF2	9	20%	**SPSLSGLKL**	PRI2	9	20%
**RPHTLNSTSM**	KMT2D	10	20%	**SVASVLLYL**	PRKDC	9	20%
**RPKGLGVFF**	VP13D	9	20%	**TPRDLAVPAAL**	PO210	11	20%
**RPNLLLGL**	PHF3, DIDO1	8	20%	**TPRPGQEL**	ZC12A	8	20%
**RPPGGHSNL**	SMBT1	9	20%	**TPRPSSPGGL**	RBG1L	10	20%
**RPRSNSAWQIYL**	MINK1	12	20%	**VPENSRPAT**	SH3L1	9	20%
**RVASPKLVM**	RTCB	9	20%	**VPNWHRDL**	RAN	8	20%
**SLASITVPL**	GGA1	9	20%	**VPRSKPLML**	ADNP	9	20%
**SPASLARTL**	TOX2	9	20%	**VPSKRQEAL**	KTN1	9	20%
**SPGGHNRPGTL**	GPS2	11	20%	**WASPPGRWL**	PTCA	9	20%
**SPISSNSHRSL**	BIRC6	11	20%	**YPRSVAVL**	PKHG1	8	20%
**SPRSSSRMEERL**	P66B	12	20%				

Supplementary Table 8: HLA class II-restricted CLL-associated antigens

Sequence	Source protein	Peptide length	Freq.	Sequence	Source protein	Peptide length	Freq.
GSSFFGELFNQNPE	CHST2	14	59%	DDHDAVLRFNGAPTANFQQDVG	SIAT1	22	27%
SGSSFFGELFNQNPE	CHST2	15	53%	DYGNFLANEASPL	VA0D1	13	27%
VQGFESATFLGYFKSG	GELS	16	51%	GNEFWSALLEKAY	CAN1/8	13	27%
FPEEFDKTSFHKVR	GNPTA	14	45%	IPGSSYTVEIFAQVG	PTPRJ	15	27%
WIGLRWTAYEKINKWT	LY75	16	41%	KGNFNYIEFTRIL	ML12A/B	13	27%
GKYFLWVVKFNSLN	GRB2	14	39%	KPGIVYASLNHSVIG	BTLA	15	27%
EDHLFRKFHYLPFLPS	DRA	16	37%	MPGPLPRSLRELHLDHNQISRVPN	FMOD	24	27%
FQVLKSLGKLAMG	SIAT1	13	37%	QQRLKSQDLELSWN	FCER2	14	27%
HHWLLFEMSRHSLE	HG2A	14	37%	RRWRFTFSHFVVDPD	I17RA	15	27%
INEFSISSFCTVVD	FMOD	14	37%	SDMFNYEEYCTANAV	SPIT2	15	27%
TGSMSIIFFLPLK	PEDF	13	37%	TDQFSGQHWLWIG	LY75	13	27%
WNFEKFLVGPDG	GPX1/3/5/6	12	37%	YPDRPGWLRYIQRTPYSDG	SGCE	19	27%
HAFFRYIDWEKLERK	KPCB	15	35%	YPRKNLFLVEVTQLTESDSGVY	FAIM3	22	27%
IPPFHPFHPFPALPENEDTQPE	APLP2	22	35%	YPRKNLFLVEVTQLTESDSGVYA	FAIM3	23	27%
KFLFVREPFERLVS	CHSTB	14	35%	AKPEASFQVWNKDSSSKNLIPR	SIAT1	22	25%
LNEDLRSWTAADTAAQITQ	HLAB, HLAC	19	35%	APIDKKGNFNYIEFTRIL	ML12A/B	18	25%
SGSSFFGELFNQNPEV	CHST2	16	35%	DGRRLAVRFTALDLGFG	LRP10	17	25%
TIQFIQSYFVTDYDPT	RRAS2	16	35%	DGTFQKWAAVVVPSGEEQ	HLAA/C/E/G/H	18	25%
ATPLLMQALPMGALPQGPMQ	HG2A	20	33%	DHAQLVAIKTLKDYNNPQ	ROR1	18	25%
DNVLYMEIRARLLPV	CECR1	15	33%	DKNLIKYDLQNLKPY	PTPRC	15	25%
ETIDWKVFESWM	HG2A	12	33%	DQFSGQHWLWIGLN	LY75	14	25%
GPSLLPIMWQLYPDG	TCL1A	15	33%	DYIALNEDLRSWTAADTAAQITQ	HLAB, HLAC	23	25%
IKDAMVATFFDIYEDG	TIP	16	33%	EDLRSWTAVDTAAQ	HLAE	14	25%
NPPPTIRWFKNDAPVVQ	ROR1	17	33%	EPDPKGIPEFWFTIFRNVD	NP1L4	19	25%
SAYKWKETLFSVMPGL	ITIH4	16	33%	EPNKKFFELVGRTFDWH	HS3S1	17	25%
TDQFSGQHWLWIGLN	LY75	15	33%	EQNEIIDDLANLVE	STX8	14	25%
VPERVYSMNPSIRLL	HS3S1	15	33%	FGLIKLDLKTKSENG	VDAC1	15	25%
AEQQRLKSQDLELSWNLNG	FCER2	19	31%	GDGTFQKWAAVVVPSGEEQR	HLAA/C/E/G/H	20	25%
DRATWKSNYFLKIIQ	RLA0, RLA0L	15	31%	GGTFKLELFLPEE	UBE2N	13	25%
DVLPKYILDFSL	CHSTE	12	31%	GKSTLINSLFLTDLYSPE	SEPT7	18	25%
EQNFQWSIYLPSSPE	IGSF3	15	31%	GKYFLWEEKFNSLNEL	GRAP	16	25%
FRSYVWDPLLIL	SYS1	12	31%	GSSFFGELFNQNPEV	CHST2	15	25%
KSTLINSLFLTDLYSPE	SEPT7	17	31%	HPAENGKSNFLNCYVSGFHPS	B2MG	21	25%
LKTIDWVAFAEIIPQ	ATP5H	15	31%	KRRLNWIQWASL	S35A5	12	25%
LPHSGDIIATVFAPL	XPR1	15	31%	LLLILRDPSERVLSDY	HS3S1	16	25%
MRMATPLLMQALPM	HG2A	14	31%	LLLWHWDTTQSLK	FCER2	13	25%
TGRFMWIKFSSDEE	NETO2	14	31%	LNLKWSRFARVVL	NRAM2	13	25%
AVRRLIWEKNLKF	CATS	13	29%	MPGPLPRSLRELHLDHNQISR	FMOD	21	25%
EQQRLKSQDLELSWNLNG	FCER2	18	29%	QQRLKSQDLELSW	FCER2	13	25%
GFMTTAFQYIIDNK	CATS	14	29%	REIDDHDAVLRFNGAPTANFQQDVG	SIAT1	25	25%
GGDKKRKGQVIQF	RL36A, RL36L	13	29%	RFSVIWQLVDRQNRR	IGSF3	15	25%
GKSTLINSLFLTDLYPE	SEPT2	17	29%	SGSSFFGELFNQNPEVF	CHST2	17	25%
GKYFLWVVKFNSL	GRB2	13	29%	SPSPQDWRDTLFYGVF	SEM4B	16	25%
IPEFWLTVFKNVD	NP1L1	13	29%	TKEFQVLKSLGKLAM	SIAT1	15	25%
LLLWHWDTTQSLKQLE	FCER2	16	29%	TPQGPPEIYSDTQFPSLQ	CDV3	18	25%
LLWHWDTTQSLK	FCER2	12	29%	VPRPYIAARFSVLPPTFHPG	PTPRS	20	25%
NKGIDSDASYPYK	CATS	13	29%	VPSRMKYVYFQNNQ	FMOD	14	25%
QPPDWLQGHYLVVRYEDL	CHST2	18	29%	AGQPLWPPVFVNL	I27RA	13	22%
TKQLFEVLHFLAEN	SL9A7	14	29%	ARLTESLFLDLLG	IL4RA	13	22%
TPKIQVYSRHPAENGKSNF	B2MG	19	29%	ARNFERNKAIKVIIAV	CCR7	16	22%
AEQQRLKSQDLELSWNLNGLQ	FCER2	21	27%	DGIIMIQTL	CD79B	9	22%
AGKYFLWVVKFNSL	GRB2	14	27%	DLEFMNEQKLNRYPA	TMM59	15	22%
AKFALNGEEFMNFD	FCGRN	14	27%	DLRSWTAVDTAAQ	HLAE	13	22%
ATPLLMQALPM	HG2A	11	27%	DQFSGQHWLWIG	LY75	12	22%

Sequence	Source protein	Peptide length	Freq.	Sequence	Source protein	Peptide length	Freq.
EDLRSWTAADTAAQITQRKWE	HLAB, HLAC	21	22%	EGQGFHILIPTIL	FAIM3	13	20%
EEVVEIDGKQVQQKD	GINM1	15	22%	EPLVVKVEEGDNAVL	CD19	15	20%
EFQVLKSLGKLAMG	SIAT1	14	22%	EQQRLKSQDLELSW	FCER2	14	20%
GDGLTYNDFLILPG	IMDH2	14	22%	EQQRLKSQDLELSWNL	FCER2	16	20%
GDGTFQKWAAVVVPSGEEQ	HLAA/C/E/G/H	19	22%	FLETHFLDEEV	FRIL	11	20%
GKKELQVSLFQTL	CUL4B	13	22%	GLGVTKQDLGPVPM	HG2A	14	20%
GPLPRSLRELHLDHNQI	FMOD	17	22%	GPLPRSLRELHLDHNQISRVPN	FMOD	22	20%
GSSLKILSKGKRGG	CXCR4	14	22%	GPPIPQNQRFIPINGYPIPPG	ROR1	21	20%
GTKVVLDDKDYFLFR	CH10	15	22%	GPPKLDIRKEEKQIMIDIFHPS	INGR1	22	20%
HFELGGDKKRKGQVIQF	RL36A, RL36L	17	22%	GVFEWEAFARGTK	PGK1	13	20%
IGVKFRNDLFKLFK	CCR7	14	22%	HAFFRYIDWEKL	KPCB	12	20%
IHEHMVITDRIENIDHLG	ITM2B	18	22%	HGNQITSDKVGRKV	FMOD	14	20%
KAVLLGATFLIDYM	PLS3	14	22%	HWLLFEMSRHSLE	HG2A	13	20%
KEIHLYQTFVVQLQDPREPR	IL2RG	20	22%	IGVKFRNDLFKLFKD	CCR7	15	20%
KFLFVREPFERLVSA	CHSTB	15	22%	IPTSSFVVDKFAGDIL	DDX60	16	20%
KQLFEVLHFLAE	SL9A7	12	22%	ISHPFFNDFTFDYD	ST14	14	20%
LLWHWDTTQSLKQLE	FCER2	15	22%	IVSIKTENTDASWNL	TM87B	15	20%
NPILYAFLGAKFKTSAQHA	CXCR4	19	22%	KGDDVKFEEFVAYLIDPH	CHSTB	18	20%
QARNFERNKAIKVIIA	CCR7	16	22%	KKVLHMDRNPYYG	GDIA/B	13	20%
QQILHSEEFLSFFD	DC1I2	14	22%	KPEASFQVWNKDSSSKNLIPR	SIAT1	21	20%
RLWAWEKFVYLDEK	TCL1A	14	22%	LATFSTDQELRFVL	IDD	14	20%
RMATPLLMQALPMGALPQGPM	HG2A	21	22%	LKNTMETIDWKVF	HG2A	13	20%
RNLKYLPFVPSRMK	FMOD	14	22%	LLLLWHWDTTQSLKQ	FCER2	15	20%
RPGLRDVAYQYVKKG	SSBP	15	22%	LMQALPMGALPQ	HG2A	12	20%
RSWTAADTAAQIT	HLAB, HLAC	13	22%	LPDQSFLWNVFQRVD	PDCD6	15	20%
SFEPPEFEIVGFT	INAR2	13	22%	LPGNATISKAGKLPYHH	PAR14	17	20%
SFKLQTKEFQVLKSL	SIAT1	15	22%	LWVVKFNSLNEL	GRB2	12	20%
SFKLQTKEFQVLKSLG	SIAT1	16	22%	MTIEPSTFLAVPT	TLR9	13	20%
SMRYFYTAVSRPGRGEPR	HLAC	18	22%	NKGIDSDASYPYKAM	CATS	15	20%
SRSYYWIGIRKIGGIW	LYAM1	16	22%	NKIFLPTIYSIIF	CXCR4	13	20%
TGSMSIIFFLPLKVT	PEDF	15	22%	NRRTFETARHNLIIN	SGCE	15	20%
VAREFGVNVFIVSVAKPIP	COCH	19	22%	NVFLRHERFERFR	TOM1	13	20%
VATMNSEEFVLVPQYA	RBG1L	16	22%	QARNFERNKAIKVII	CCR7	15	20%
VGSFVGSGLLAL	S15A4	12	22%	QQRLKSQDLELSWNLN	FCER2	16	20%
VGYVDDTLFVRFDSD	HLAB	15	22%	RKLFSSHRFQVII	HVCN1	13	20%
VKKMMKDNNLVRH	AT2B1/2/3/4	13	22%	RPAGDRTFQKWAAVVVPSGEEQRYT	HLAB	25	20%
WNFEKFLVGPD	GPX1/3/5/6	11	22%	RVTLKQYPRKNLFLV	FAIM3	15	20%
AGLGRAYALAFAERGAL	DHB4	17	20%	SDGLNSLTYQVLDVQRYPLY	B4GT1	20	20%
APLDFRGMLRKLFS	HVCN1	14	20%	SDLSFSKDWSFYLL	B2MG	14	20%
ASILATAANLLRHYP	TM127	15	20%	SELIKIIRRRLQLNAN	MLP3B/2	16	20%
AVGYVDDTQFVRFDSDA	HLAA/B/C	17	20%	SFFKISYLTFLPS	DQA2	13	20%
AYDGKDYIALNEDLRSWTA	HLAA/C/E/G/H	19	20%	SPNELVDDLFKGAKEHG	NSF1C	17	20%
DGLNSLTYQVLDVQRYPLY	B4GT1	19	20%	SPPPEFSFNTPGKNVNPV	ZNT6	18	20%
DGQKFSVTAYSEWIE	TMEM2	15	20%	SPSPGVYRLFIQNVAVQDSGTY	IGSF3	22	20%
DGTFQKWAAVVVPSGE	HLAA/C/E/G/H	16	20%	SSWYEVDSFTPFR	INAR1	13	20%
DGTFQKWAAVVVPSGQ	HLAA/B/C	16	20%	TSADLFLDQTELAAIN	TICN2	16	20%
DGTFQKWAAVVVPSGQE	HLAA/B/C	17	20%	VARLSRDATFHYGEQ	IGSF3	15	20%
DLRSWTAADTAA	HLAB/C/G	12	20%	VDDTQFVRFDSDAASQRMEPRAPWI	HLAA	25	20%
DNKGIDSDASYPY	CATS	13	20%	VGQFIQDVKNSRSTD	CAND1	15	20%
DPTLDHHWHLWKKTYGKQYKEKNE	CATS	24	20%	VGRKVFSKLRHLER	FMOD	14	20%
DQPTIRKENFNNVP	CHP3	14	20%	VLRFNGAPTANFQ	SIAT1	13	20%
DRLWAWEKFVYLDE	TCL1A	14	20%	VNLIEKVASYGVKPRYG	CFAB	17	20%
DRLWAWEKFVYLDEK	TCL1A	15	20%	VPRKVIIDDQLPVDHKG	CAN7	17	20%
DYGIVADLFKVVP	ETFA	13	20%	VPSRMKYVYFQNNQITSIQ	FMOD	19	20%
EDYLSVVLNQL	ALBU	11	20%	YKIVNFDPKLLE	GL8D1	12	20%
